# Facial palsy reveals the sensorimotor contribution to facial-emotion recognition

**DOI:** 10.1073/pnas.2608511123

**Published:** 2026-09-11

**Authors:** Paola Sessa, Arianna Schiano Lomoriello, Thomas Quettier, Antonio Maffei, Sara Costa, Marta Nichele, Pier Francesco Ferrari

**Affiliations:** ^a^ Department of Developmental Psychology and Socialisation, University of Padova, Padova 35131, Italy; ^b^ Padova Neuroscience Center, Department of Developmental Psychology and Socialisation, University of Padova, Padova 35129, Italy; ^c^ Department of Medicine and Surgery, University of Parma, Parma 43126, Italy; ^d^Independent Physiotherapist, Padova 35143, Italy; ^e^ Social Neuroscience and Comparative Development, Social Neuroscience and Comparative Development, Institut des Sciences Cognitives Marc Jeannerod, CNRS/Université Claude Bernard Lyon 1, Bron Cedex 69675, France

**Keywords:** embodied cognition, facial paralysis, emotion recognition, sensorimotor calibration, Moebius syndrome

## Abstract

Although recognizing emotions from facial expressions appears effortless, the field is divided between vision-based accounts, which posit matching to learned visual templates, and embodied accounts, which posit recruitment of facial motor circuits. We propose an ambiguity-gated, developmentally calibrated architecture in which the recognition system draws on sensorimotor information primarily when visual evidence is insufficient, with early motor experience proposed to shape the threshold and gain of this contribution. We tested this model across three studies (N = 185) integrating dynamic prototypical and nonprototypical expressions, congenital (Moebius syndrome) and acquired facial palsy, and standardized severity grading (Sunnybrook Facial Grading System; SFGS). Neurotypical adults (N = 117) confirmed a robust prototypicality-dependent cost on recognition. Both congenital (N = 15) and acquired (N = 19) cohorts showed severity–performance coupling for nonprototypical expressions, consistent with shared reliance on sensorimotor information when visual evidence is insufficient; only the Moebius cohort showed this coupling also for prototypical expressions, consistent with undercalibrated visual templates following lifelong atypical facial motor experience. Exploratory electroencephalography (EEG) revealed reduced sensorimotor–face-network functional connectivity in Moebius, consistent with altered cross-route integration. Reanalysis of an independent dataset of ultra-ambiguous static morphs replicated severity–performance coupling and revealed Moebius–control mean differences only under high perceptual demand. Together, the findings provide strong behavioral support for an ambiguity-gated, developmentally calibrated architecture and motivate direct tests of how developmental motor experience shapes when—and how strongly—the recognition system draws on sensorimotor information.

## Significance

How do we recognize emotions from other people’s faces? A long-standing debate opposes two answers: that we match faces to learned visual patterns, or that we covertly draw on our own facial movements. Facial palsy provides a natural experiment. It removes facial movement without artificial interference, and contrasting people paralyzed from birth (Moebius syndrome) with those paralyzed later in life isolates the role of early motor experience. Grading residual movement, we found that it predicted recognition of ambiguous expressions, but not of clear ones—except in those affected from birth, where it predicted recognition even of clear expressions. The result turns a long-standing either-or debate into a testable account of when and why visual and sensorimotor information each contribute.

Facial expressions are our fastest readout of other minds, yet the mechanisms supporting this efficiency remain debated. Vision-centric accounts posit that a rapid, largely feed-forward sweep through visual cortex matches incoming configurations to learned templates (1, 2). Embodied accounts argue that sensorimotor recruitment—including subtle activation of one’s own facial musculature—supplies generative constraints for extracting emotional meaning (3, 4). We propose that these accounts are not mutually exclusive but interact through an ambiguity-gated, developmentally calibrated mechanism. When visual evidence is strong, recognition proceeds within visual circuits and stored perceptual templates. As perceptual uncertainty increases, the recognition system progressively draws on sensorimotor information to support the decision. This use of sensorimotor information operates online, trial by trial, whenever the visual route alone cannot resolve the stimulus at hand. It rests, however, on a slower foundation laid down over early life: through motor experience, the visual templates against which expressions are weighed are progressively calibrated. Well-calibrated templates resolve most expressions on visual grounds alone; where calibration is incomplete, even clear expressions retain residual ambiguity. Early motor experience thus acts on recognition over development, and the templates it leaves behind govern, in the moment, how far the visual route can carry recognition on its own—and, correspondingly, how much the sensorimotor route must supply.

In healthy participants, perturbing facial movements yields heterogeneous effects on emotion recognition (5–11), likely because many studies relied on prototypical expressions that drive performance to the ceiling, masking small sensorimotor contributions (5, 6). When the task is genuinely hard—with ambiguous, nonprototypical, or morphed expressions—restricting facial feedback impairs discrimination and increases EMG signatures of sensorimotor support (4, 12). Crucially, this evidence leaves a central question unresolved: what determines the threshold at which sensorimotor information enters the recognition decision, and the gain with which it does so? Here, we provide evidence consistent with the view that this tuning is shaped by developmental history and residual motor capacity, offering a candidate explanation for individual variability and for the mixed results in the literature.

Facial palsy provides a natural experiment: it removes specific facial motor output and proprioceptive feedback without artificial interference. Congenital cases (e.g., Moebius) often show preserved accuracy on easy emotion tasks despite absent motor output (2, 13), while neuroimaging suggests compensatory reorganization (14). In acquired palsy, deficits vary as a function of task demands and stimulus ambiguity (15, 16). Critically, congenital and acquired cohorts have never been examined jointly, despite offering a unique testbed for how early calibration vs. late degradation shape the balance between visual and sensorimotor routes. A further limitation concerns palsy severity: congenital palsy is frequently treated as all-or-none, obscuring residual capacity, and standardized grading in acquired palsy has only recently been adopted [e.g., Sunnybrook Facial Grading System (SFGS); 16]. Modeling severity continuously is crucial because residual capacity likely sets an individual’s position relative to the threshold at which sensorimotor support becomes functionally necessary.

Prior work has probed one axis at a time—ambiguity or developmental motor history—while largely ignoring graded severity. We address this gap by proposing an ambiguity-by-development framework that explicitly crosses: i) stimulus ambiguity (prototypical vs. ambiguous expressions); ii) residual motor capacity; and iii) developmental timing (congenital vs. acquired) alongside neurotypical controls.

This design operationalizes the ambiguity-gated, developmentally calibrated architecture proposed above, which rests on two constructs from which the design choices follow directly (Fig. 6):

i) The ambiguity-gated model: A dual-route architecture in which a visual template-matching route operates in parallel with a sensorimotor reconstruction route, with the relative weighting of the two routes determined by the perceptual conditions of the input. When stimuli map cleanly onto stored expressive prototypes, the visual route is sufficient on its own: input is matched to a stored template, and a recognition decision follows. As configurations depart from those prototypes (i.e., through atypical action-unit combinations, competing configural cues, or reduced intensity), the visual route becomes progressively insufficient, and the recognition system places greater weight on the sensorimotor route, which infers expressive meaning from the motor and somatosensory consequences of internally simulating the perceived configuration in one’s own facial musculature. We term this moment-to-moment engagement on-demand sensorimotor bootstrapping (Fig. 6, gate within each panel). The gate captures this conditionality: sensorimotor information enters the recognition decision when, and insofar as, the visual route alone fails to disambiguate the input. Whether this dependence is graded and continuous or threshold-like is an empirical question the present data do not resolve; we adopt the gate framing as a tractable simplification that does not presuppose the functional form. Fig. 6 follows the same convention. The model is the architectural locus at which the long-standing opposition between vision-centric and embodied accounts becomes reconcilable, by specifying when each route contributes rather than asserting that either is necessary in general.
ii) The ambiguity-by-development framework: A 2 × 2 conceptual scheme crossing stimulus ambiguity (prototypical vs. nonprototypical) with developmental timing of motor impairment (congenital vs. acquired). The framework follows from a single premise: the visual templates on which recognition relies are themselves calibrated by an individual’s facial motor experience over development—a process we term early sensorimotor bootstrapping (Fig. 6, T1 and A1). Early sensorimotor bootstrapping and on-demand bootstrapping are the developmental and online faces of sensorimotor contribution: the first shapes the templates over early life, the second supplements the visual route in the moment when those templates prove insufficient. In congenital absence of motor experience, the route is calibrated atypically across the lifespan, with consequences detectable for both prototypical and nonprototypical stimuli. In acquired impairment, the route is typically calibrated prior to onset, and the consequences of subsequent motor loss manifest specifically where the visual route is insufficient, i.e., for nonprototypical stimuli, where the sensorimotor route would otherwise have supplied disambiguating input. The discriminative power of the design lies in the joint manipulation of the two factors: the same residual motor capacity carries different developmental meanings across etiologies, and the same stimulus class probes different mechanisms across cohorts. This expected asymmetric pattern is difficult to accommodate within purely visual or uniformly embodied accounts, but follows naturally from a framework in which sensorimotor contributions depend jointly on ambiguity and developmental history.

Two testable predictions follow from these two constructs. The first concerns within-cohort variability with an etiology-specific pattern: in each clinical cohort, recognition accuracy should scale continuously with residual motor capacity, but the conditions under which this scaling appears should differ across etiologies. In congenital palsy (Moebius), where lifelong absence of motor experience leaves visual templates undercalibrated, severity should predict recognition for both prototypical and nonprototypical expressions; in acquired palsy, where preonset calibration left visual templates intact, severity should predict recognition selectively for nonprototypical expressions. The cross-etiological dissociation is the diagnostic behavioral pattern predicted by the developmental-calibration account. The second concerns between-group differences: mean recognition accuracy should diverge between clinical cohorts and neurotypical controls when stimulus ambiguity exceeds the threshold at which the visual route alone becomes insufficient. The within-cohort prediction does not depend on where this threshold sits and can be tested directly with dynamic, naturalistic expressions in our main behavioral studies (Studies 2 and 3). The between-group prediction, by contrast, is threshold-dependent: whether our dynamic naturalistic stimuli fall above or below the visual-sufficiency threshold is itself an empirical question, which we address by complementing the main studies with reanalysis of an independent dataset of ultra-ambiguous static morphs (Boundary checks, *Results*) to probe the suprathreshold regime directly.

Two operational choices follow directly. First, residual motor capacity is indexed by SFGS, a clinician-rated instrument that quantifies preserved facial-nerve function across resting symmetry, voluntary movement, and synkinesis on a continuous scale. SFGS captures the substrate available to the sensorimotor route, i.e., the more motor function remains, the more the route can contribute, and is therefore the within-cohort grading variable on which the developmental dissociation is tested. Critically, the same numerical SFGS value carries distinct developmental meanings across etiologies: in Moebius, it approximates lifelong sensorimotor input, including any sensitive-period calibration that did or did not occur; in acquired palsy, it indexes postcalibration motor preservation following an otherwise typical developmental trajectory. Second, the diagnostic test of the architecture is the severity–performance coupling, i.e., the within-cohort correlation between SFGS and recognition accuracy. If the sensorimotor route contributes in proportion to the motor capacity available to it, individuals with greater preserved capacity should show better recognition under conditions in which that route is functionally relevant. The conditions under which this coupling emerges (or does not), across stimulus types and across etiologies, constitute the diagnostic empirical signature of sensorimotor contribution to recognition.

These predictions translate into three operational hypotheses for testing:

1. H1: *Ambiguity impairs performance*. Neurotypical adults should perform worse on nonprototypical than on prototypical expressions.
2. H2: *Severity–performance coupling with an etiology-specific pattern*. Recognition accuracy should scale with residual motor capacity. In Moebius, greater severity should predict poorer recognition for both prototypical and nonprototypical expressions (lack of early calibration). In acquired palsy, severity effects should emerge selectively for nonprototypical expressions, since prototypical recognition is supported by preonset calibrated templates, yielding cross-etiological convergence for ambiguous stimuli and divergence for prototypical ones.
3. H3: *Exploratory neural characterization of sensorimotor–visual integration (Moebius).* EEG μ-band ERD (an event-related desynchronization in the 8 to 13 Hz band over central sensorimotor sites indexing the modulation of local sensorimotor activity during expression observation), and functional coupling between sensorimotor cortex and the core face network (indexing cross-route integration hypothesized by the dual-route architecture) should be attenuated relative to controls.

To test these hypotheses, we ran three coordinated studies (total N = 185).

The goal of Study 1 was to establish the ambiguity-gated baseline in neurotypical adults, validating the ambiguity manipulation for the subsequent testing in clinical cohorts. To this aim, we used two corpora that operationalize opposite poles of an expressive-prototypicality continuum: the Amsterdam Dynamic Facial Expression Set (ADFES; 17), in which trained actors produce maximally clear, FACS-validated configurations of the basic emotions, and the Jerusalem Facial Expressions of Emotion (JeFEE; 18), in which nonactor expressers produce spontaneous expressions whose action-unit patterns are configurally noncanonical relative to those prototypes.

The goal of Study 2 was to test the architecture’s predictions in congenital facial palsy, where lifelong absence of facial motor experience compromises sensorimotor calibration; we combined behavioral testing with high-density EEG in 15 individuals with Moebius syndrome and matched controls—the largest neurophysiologically characterized Moebius cohort to date. The goal of Study 3 was to extend the test to acquired facial palsy, where preonset typical calibration is followed by motor loss; we ran the same design in 19 adults with acquired facial paralysis and matched controls. Across Studies 2 and 3, a physiotherapist, blind to hypotheses and to the participant’s performance on the experimental task, assessed palsy severity with the SFGS, while we controlled for potential confounds (autistic traits, alexithymia, face-identity skills, mid-level vision, depressive symptoms). A priori power analyses confirmed adequate power for the expected behavioral effects (H1, H2: confirmatory); neurophysiological tests remain exploratory. To maximize robustness, we integrated frequentist, Bayesian, and bootstrap statistics.

By jointly modeling stimulus ambiguity, palsy severity, and developmental timing, our work adjudicates between purely visual, uniformly embodied, and developmentally agnostic accounts of facial-emotion recognition, and offers proof of concept for an ambiguity-gated, developmentally calibrated architecture in which the recognition system’s reliance on sensorimotor information is conditional on perceptual demand and shaped by developmental motor experience, and establishes a behavioral platform for future mechanistic tests of this architecture.

## Results

We tested the ambiguity-gated model using a hierarchical strategy comprising confirmatory behavioral analyses, planned exploratory neural analyses, and additional exploratory tests (*Materials and Methods* and *SI Appendix*, sections 1.5 and 1.6). We first validated the ambiguity manipulation in neurotypical adults (n = 117; Study 1), then tested the core behavioral prediction in the two clinical cohorts (congenital facial palsy, i.e., Moebius syndrome, Study 2, and acquired facial palsy, Study 3), and examined EEG markers in the Moebius cohort (Study 2).

### Ambiguity Manipulation (H1).

**Fig. 1.**
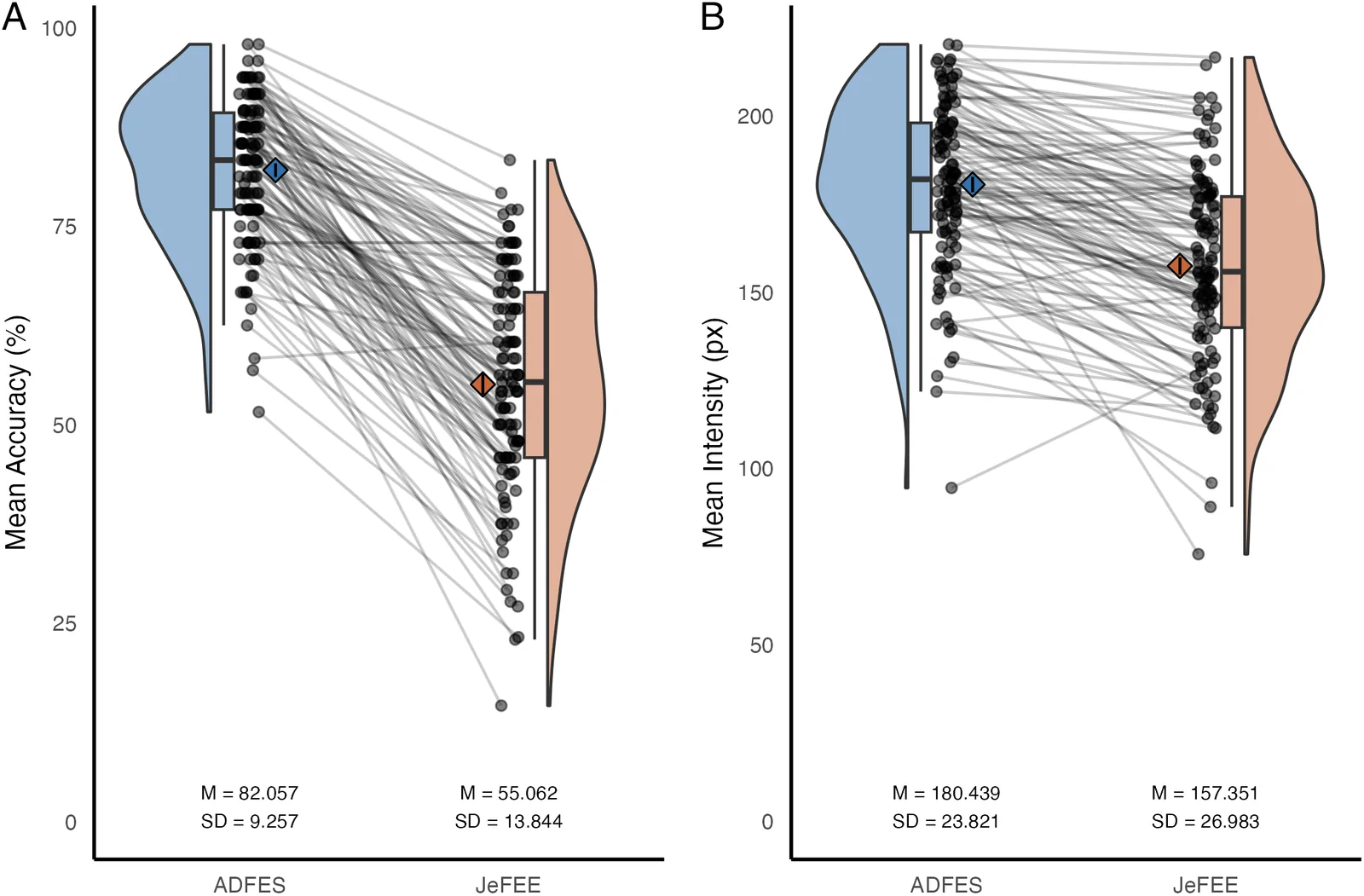
Recognition accuracy (*A*) and perceived intensity (*B*) for prototypical (ADFES) and ambiguous (JeFEE) facial expressions in the reference cohort (n = 117). Violin plots show individual mean scores with boxplots indicating interquartile range and median; black diamonds denote group means ± SE. Both measures were significantly higher for ADFES than JeFEE. These distributions provide reference benchmarks for clinical comparisons.

The goal of Study 1 was to establish the ambiguity-gated baseline in neurotypical adults, validating the ambiguity manipulation for the clinical contrasts that follow. ADFES (prototypical) and JeFEE (nonprototypical) anchored opposite poles of an expressive-prototypicality continuum. The architecture-specific prediction was that nonprototypical expressions would impose a measurable recognition cost (H1). Without this benchmark, any difference between ADFES and JeFEE performance observed in Studies 2 and 3 would be ambiguous: a clinical cohort scoring lower on JeFEE could reflect either etiology-related vulnerability to nonprototypical expressions or simply the intrinsic difficulty asymmetry between the two corpora. We therefore contrasted ADFES and JeFEE within-subject in 117 adults recruited online, a design that separates stimulus-level from individual variance. Recognition accuracy was sharply asymmetric—ADFES near ceiling (82.06% ± 9.26%), JeFEE markedly lower (55.06% ± 13.84%)—calibrating the prototypicality manipulation and benchmarking the clinical contrasts that follow. This prototypicality effect was remarkably robust, explaining 12% of variance in accuracy (β = − 1.38, SE = 0.04, z = − 30.05, *P* < 0.001, d = 0.76, BF_10_ = 2.82 × 10^218^). Perceived intensity showed a parallel pattern (ADFES: M = 180.44 ± 23.82; JeFEE: M = 157.35 ± 26.98; F(1,116) = 154.01, *P* < 0.001, η_p_^2^ = 0.57, BF_10_ = 4.6 × 10^19^). These performance bands (accuracy: ADFES 51.59 to 97.92%, JeFEE 14.58 to 83.33%) establish essential benchmarks for interpreting subsequent clinical findings (Fig. 1).

### Severity–Performance Coupling in Congenital Palsy (H2a).

**Fig. 2.**
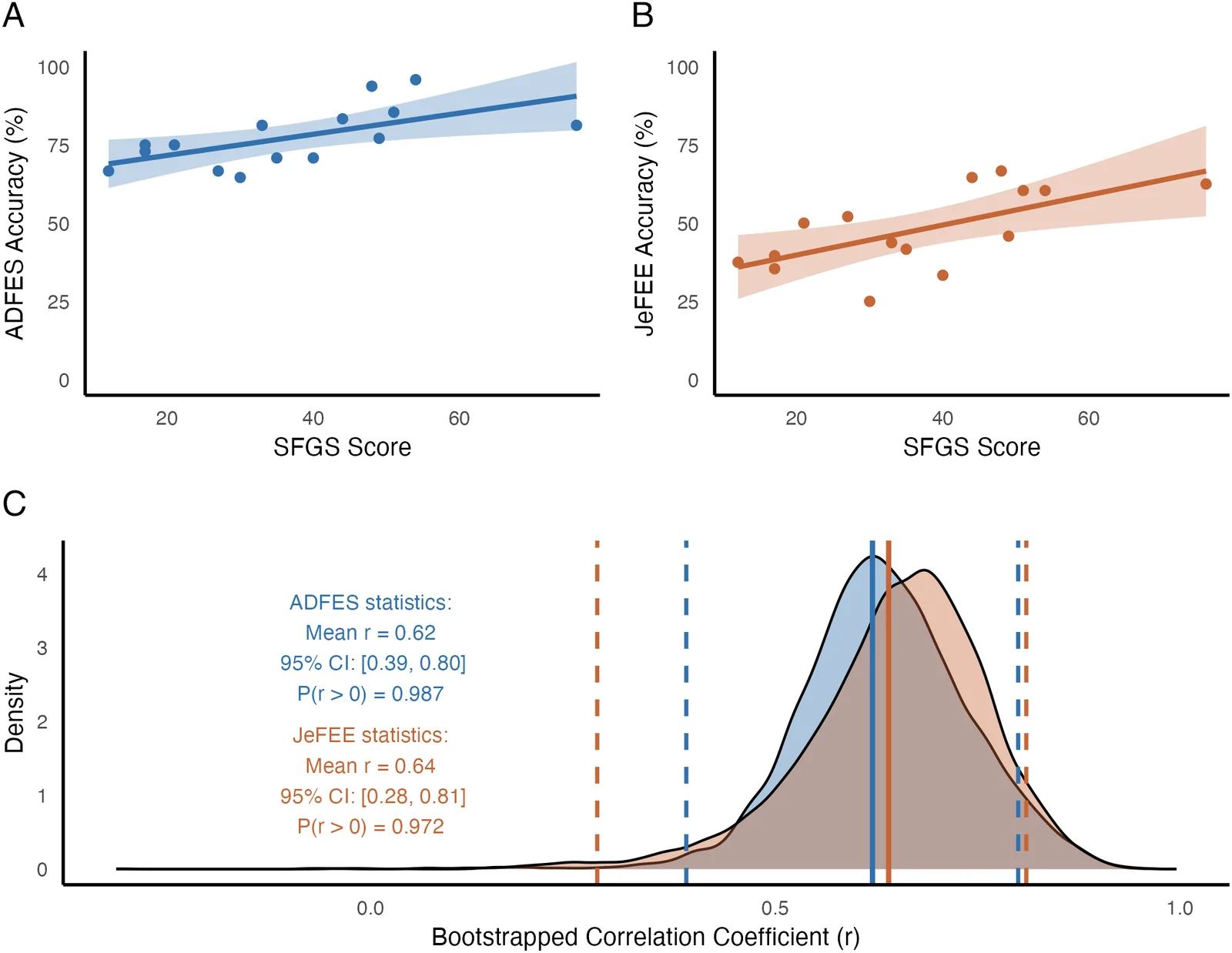
Relationship between facial paralysis severity and emotion recognition in Moebius syndrome. (*A* and *B*) SFGS scores vs. recognition accuracy for ADFES and JeFEE expressions, revealing significant positive correlations for both expression types. (*C*) Bootstrapping (10,000 iterations) confirms robust motor–recognition associations with 95% BCa CIs excluding zero. Leave-one-out cross-validation demonstrates meaningful generalization (JeFEE: R^2^_CV_ = 0.28; ADFES: R^2^_CV_ = 0.10).

**Fig. 3.**
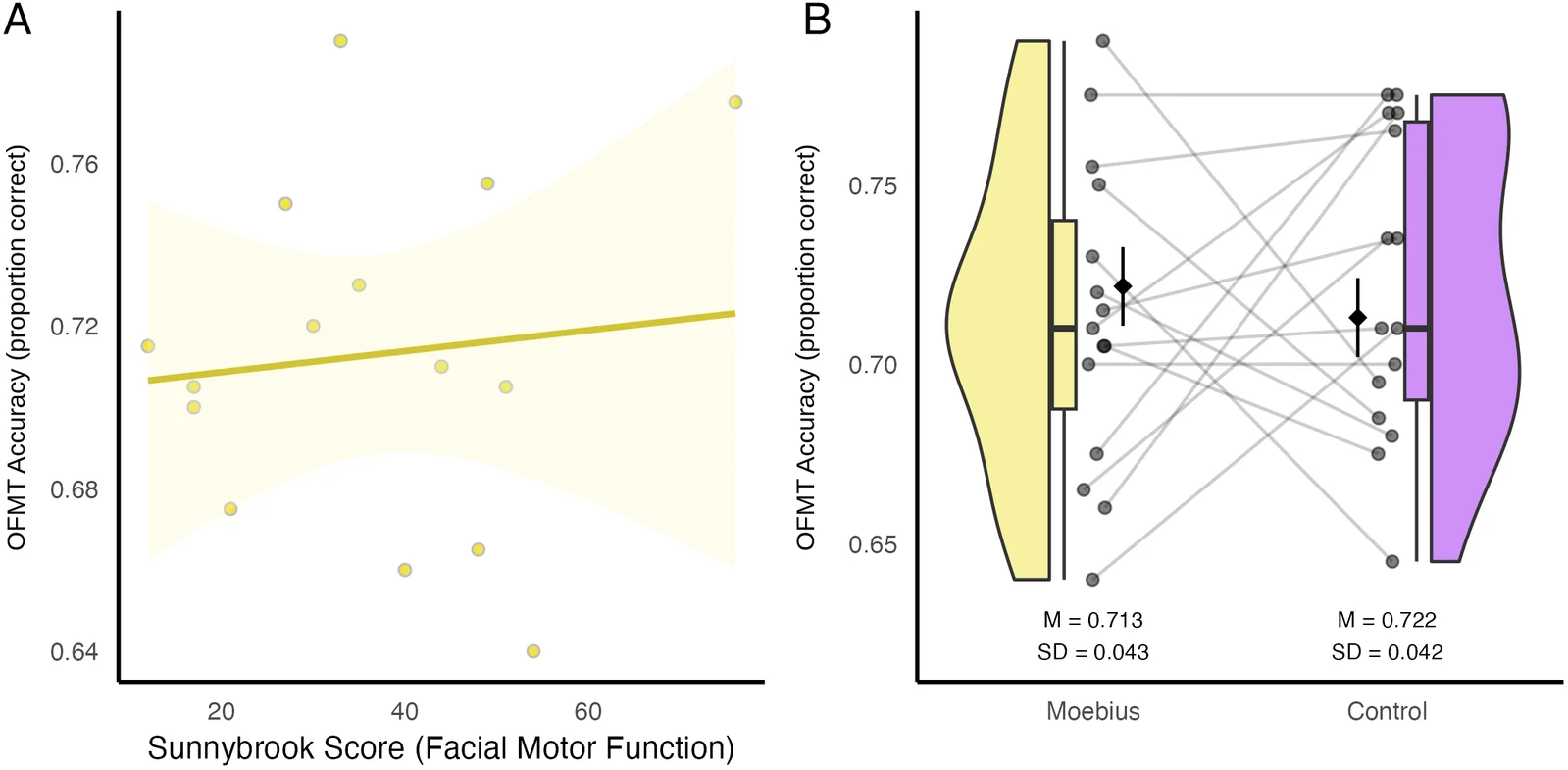
Face-identity recognition and facial paralysis severity in Moebius syndrome. (*A*) SFGS scores vs. Oxford Face Matching Test (OFMT) performance show no association (r = 0.105, *P* = 0.71, BF_01_ = 1.78). (*B*) OFMT scores for Moebius and controls show no group difference (*P* = 0.58), confirming that face-identity recognition is preserved and independent of facial motor function.

The goal of Study 2 was to test whether congenital facial palsy shows the severity–performance pattern predicted if early motor experience contributes to calibration of the sensorimotor route. The architecture-specific prediction was that in Moebius, severity–performance coupling should emerge for *both* prototypical and nonprototypical expressions—the behavioral pattern expected if visual templates are less well calibrated following lifelong atypical facial motor experience. The prior literature has treated congenital palsy as all-or-none, ignoring the residual motor variability that the architecture’s within-cohort prediction requires. We therefore tested 15 Moebius participants and 15 matched controls, scoring palsy severity continuously with the SFGS. SFGS scores correlated robustly with performance on JeFEE expressions (r = 0.64, 95% BCa CI [0.28, 0.81], *P* = 0.009, q = 0.014, BF_10_ = 6.09) and comparably for ADFES expressions (r = 0.62, 95% BCa CI [0.39, 0.80], *P* = 0.014, q = 0.014, BF_10_ = 4.78). Leave-one-out cross-validation confirmed predictive generalization (JeFEE: R^2^_CV_ = 0.28; ADFES: R^2^_CV_ = 0.10; Fig. 2). Model projections illustrate the range: a participant with severe paralysis (SFGS = 20) was predicted to score 40% on JeFEE and 72% on ADFES, whereas a mildly affected individual (SFGS = 80) would reach 69% and 92%, respectively. Importantly, face-identity processing (Oxford Face Matching Test) showed no association with paralysis severity (r = 0.105, *P* = 0.71, BF_01_ = 1.78; Fig. 3), confirming that the motor–recognition link is specific to emotional expressions.

### Severity–Performance Coupling in Acquired Palsy (H2b).

**Fig. 4.**
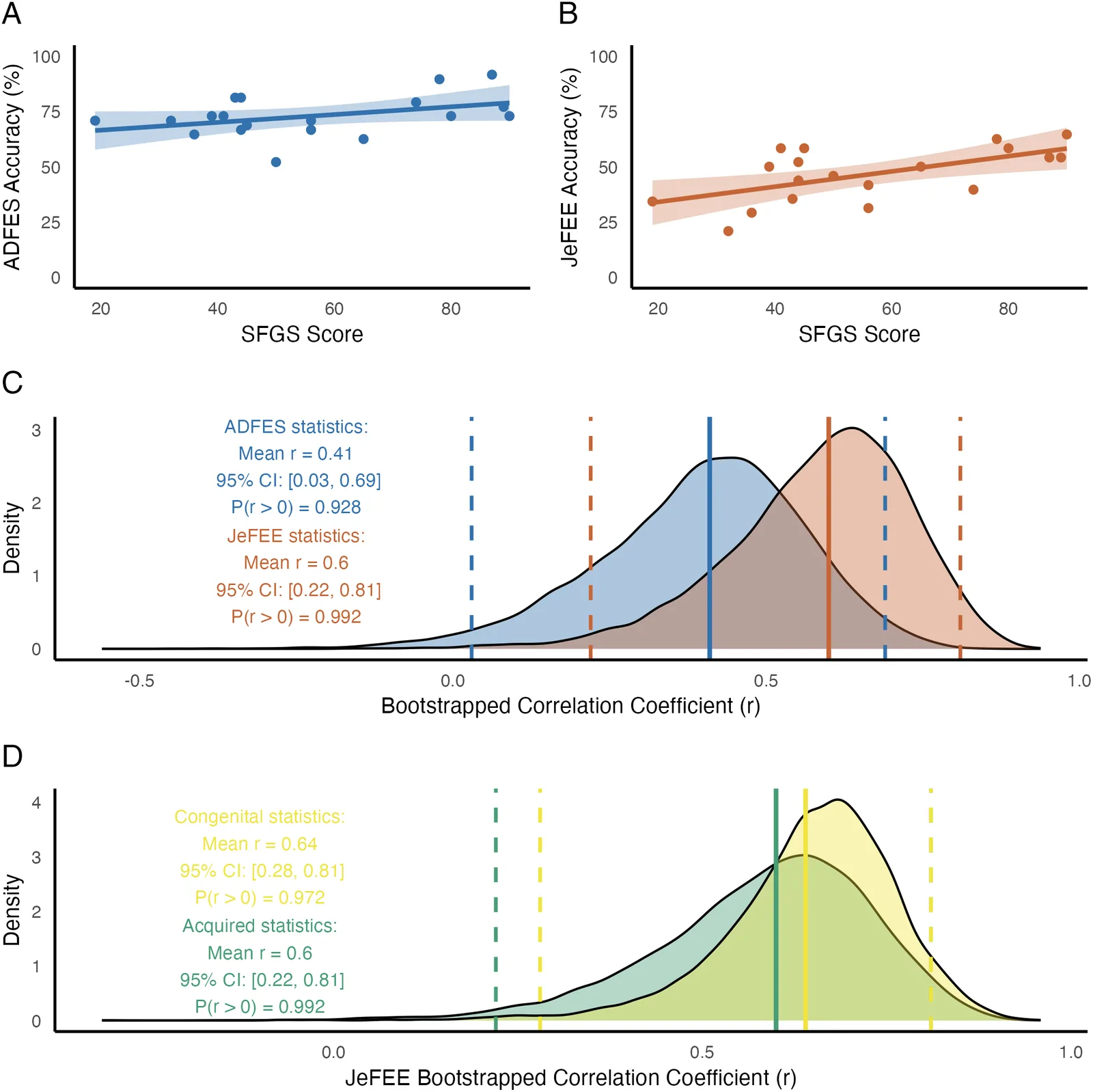
Severity–performance coupling in acquired palsy and cross-cohort comparison. (*A* and *B*) SFGS scores correlate significantly with JeFEE but not ADFES recognition. (*C*) Bootstrapping distributions confirm this dissociation. (*D*) Cross-cohort comparison reveals similar JeFEE correlation magnitudes across etiologies (r = 0.60 vs. 0.64; Fisher Δz = 0.08, *P* = 0.84) with overlapping CIs, indicating that motor–perception coupling for ambiguous expressions generalizes across etiologies while the ADFES pattern distinguishes congenital from acquired cases.

The goal of Study 3 was to test whether typical preonset calibration of the sensorimotor route insulates prototypical recognition from later motor loss while leaving nonprototypical recognition exposed. The architecture-specific prediction was that, in acquired palsy, severity–performance coupling should emerge selectively for nonprototypical expressions, with prototypical recognition preserved across the severity range, since preonset-calibrated visual templates may remain sufficient for high-signal expressions, making the sensorimotor contribution most load-bearing when the visual route is insufficient. Paired with Study 2, the design completes a developmental contrast the literature has lacked: cross-etiological convergence for ambiguous stimuli would index a shared reliance on sensorimotor information when the visual route is insufficient, whereas divergence for prototypical stimuli would identify the route’s calibration history as the dimension that differs. We tested 19 adults with acquired facial paralysis and matched controls, with continuous SFGS scoring. SFGS scores correlated strongly with JeFEE performance (r = 0.60, 95% BCa CI [0.22, 0.81], *P* = 0.007, q = 0.014, BF_10_ = 8.10), whereas the ADFES association was weaker and did not survive false-discovery control (r = 0.41, 95% BCa CI [0.03, 0.69], *P* = 0.086, BF_10_ = 1.53). Leave-one-out cross-validation reinforced this dissociation (JeFEE: R^2^_CV_ = 0.22; ADFES: R^2^_CV_ = − 0.02; Fig. 4 *A* and *B*).

Crucially, these associations were not accounted for by broader affective or perceptual factors: exploratory mediation analyses showed that alexithymia, autistic traits, depressive symptoms, and perceived intensity did not mediate the SFGS–accuracy relationship, with negligible indirect effects and unchanged direct associations in both Moebius and acquired groups (*SI Appendix*, sections 2.1.2 and 2.2.2).

### Cross-Etiological Convergence for Nonprototypical Expressions.

When expression configurations are nonprototypical and visual evidence is less decisive, the framework predicts that recognition should benefit from whatever sensorimotor information is available, regardless of how that capacity was developmentally calibrated. The architecture, therefore, predicts severity–performance coupling for JeFEE expressions in both Moebius and acquired palsy, with comparable magnitude. For JeFEE expressions, both groups showed significant motor–emotion correlations (Moebius: r = 0.64; Acquired: r = 0.60), with overlapping CI. A Fisher z-test confirmed no significant difference (Δz = 0.08, *P* = 0.84; Fig. 4*D*), supporting cross-etiological convergence for nonprototypical expression processing.

### Cross-Etiological Divergence for Prototypical Expressions.

For prototypical expressions, the framework predicts a different pattern. If visual templates were calibrated before motor loss, as in acquired palsy, prototypical recognition should remain relatively insulated from residual motor capacity. If early calibration was atypical, as in Moebius, even prototypical expressions may retain enough effective uncertainty for residual motor capacity to predict performance. The architecture, therefore, predicts a divergence—severity–performance coupling in Moebius, where templates are undercalibrated due to lifelong absence of motor experience, but absence of severity–performance coupling in acquired palsy, where preinjury calibration left templates intact and the visual route sufficient. For ADFES expressions, Moebius patients showed a moderate positive correlation (r = 0.62, BF_10_ = 4.78), whereas acquired palsy patients showed a weaker, nonsignificant association (r = 0.41, BF10 = 1.53). To rule out confounding by palsy severity (mean SFGS = 36.93 vs. 56.21), we extracted severity-matched subgroups spanning the shared SFGS range (19 to 76; Moebius n = 12, acquired n = 14). In matched subgroups, Moebius patients retained a positive correlation (r = 0.57), whereas the acquired group showed no association (r = −0.03). A bootstrap analysis (10,000 iterations) showed that 98.51% of resampled Δr values were positive (median Δr = 0.65; 95% CI [0.06, 1.22]), indicating that this divergence reflects a developmentally rooted dissociation rather than mere differences in severity.

### Consistency of the Coupling Across Emotion Categories.

A leave-one-emotion-out analysis confirmed that the severity–performance coupling was not driven by any single emotion category. Excluding each of the six expression categories in turn and recomputing the aggregate from the remaining five, the SFGS–accuracy correlation remained positive in every iteration and across both cohorts and stimulus sets (*SI Appendix*, section 2.6). This indicates that aggregate recognition accuracy reflects a broadly shared component of facial-expression recognition rather than a heterogeneous or mutually canceling emotion-specific profile.

### Neural Markers (H3, Exploratory).

**Fig. 5.**
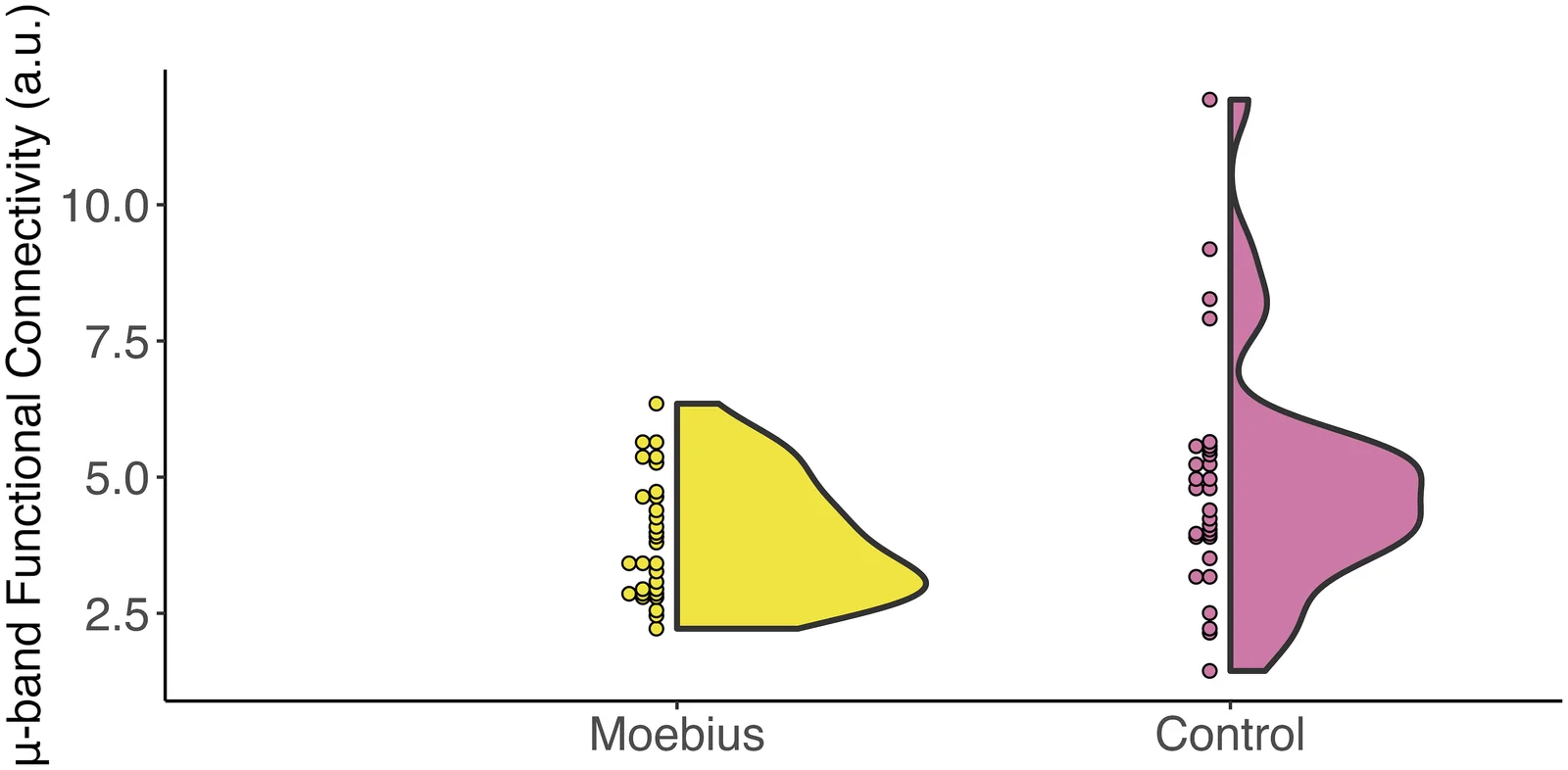
μ-band functional connectivity between sensorimotor cortices and the core face-processing system during observation of facial expressions. The figure shows the full distribution and the individual data for each participant in Moebius and control groups. Controls showed significantly higher connectivity, consistent with altered sensorimotor–visual integration in congenital facial palsy.

Sample size constraints in Study 2 (N = 15 Moebius participants) preclude condition-by-group splits of neural markers; a finer-grained, condition-specific characterization of these markers requires a larger neurophysiologically characterized cohort and is the focus of dedicated follow-up work. We therefore analyzed μ-band ERD and functional coupling as global, exploratory markers of sensorimotor system organization, asking whether congenital facial palsy is associated with altered local sensorimotor responsiveness or altered integration with face-processing regions. The two markers index complementary aspects of system organization. μ-band ERD reflects the global capacity of the sensorimotor cortex to activate during expression observation, whether the local circuitry responds to observed faces at all. Functional coupling between sensorimotor cortex and core face-processing regions indexes the integration capacity between the two routes, namely the infrastructural extent to which sensorimotor activity can be communicated to face-processing networks, setting the upper bound on the sensorimotor contribution to recognition. Such integration is the pathway through which sensorimotor activity could become relevant to the recognition decision. μ-band ERD did not differ between Moebius participants and controls (Group: F_1,56_ = 0.07, *P* = 0.79; Region: F_1,56_ = 2.40, *P* = 0.13; interaction: F_1,56_ = 0.03, *P* = 0.87), suggesting that the local activation capacity of sensorimotor cortex is preserved. Connectivity between sensorimotor cortices and core face-processing regions, by contrast, was significantly reduced (t_43_ = 2.22, *P* = 0.03, d = 0.57, BF_10_ = 1.99; Fig. 5). The dissociation, while exploratory, is theoretically suggestive: it points to integration between sensorimotor and face-processing networks as a candidate locus where developmental motor experience may leave its functional trace, consistent with the architectural geometry of the dual-route account.

### Between-Group Comparisons.

The architecture allows two regimes at the level of cohort means. Below the threshold at which the visual route alone is insufficient, calibrated visual information sustains average performance, and cohort means converge, leaving within-cohort severity grading as the diagnostic signature. Above the threshold, the visual route fails systematically, and between-group separation emerges. Whether our naturalistic dynamic stimuli—including the JeFEE corpus—would push uncertainty above or below this threshold was an empirical question. The within-cohort severity–performance coupling reported above bears on the architecture independently of where the threshold sits; the between-cohort signature, by contrast, depends on it. To probe the suprathreshold regime directly, we therefore reanalyzed an independent dataset of ultra-ambiguous static morphs from previous work (reported below). Across all cohorts, ADFES expressions were identified far more readily than JeFEE expressions (d = 0.65 to 0.76). Neither Moebius nor acquired palsy patients differed from matched controls in mean accuracy (Moebius *P* = 0.95; acquired *P* = 0.18), and no Group × Prototypicality interaction approached significance (Moebius *P* = 0.81; acquired *P* = 0.887). The pattern suggests that the JeFEE regime remained below the threshold for reliable cohort-level separation: cohort means converge while severity-graded within-cohort variability remains the diagnostic behavioral signature, consistent with the architecture under subthreshold conditions.

### Boundary Checks.

Two complementary tests probe regimes outside those sampled by the main behavioral studies. a) As anticipated above, the suprathreshold regime, i.e., where the visual route is systematically insufficient, was probed by reanalyzing an independent dataset of static, ultra-ambiguous morphed faces (19). This reanalysis showed both patient–control separation and within-Moebius severity–performance coupling under such high-demand conditions (*SI Appendix*, sections 2.4 and 2.5), consistent with the expected between-group signature in a higher-demand regime. b) The architecture also predicts that a transient reduction of facial feedback should impair recognition under demand. An exploratory pilot using hardening masks found lower recognition accuracy in masked vs. unmasked participants (*P* < 0.05), consistent with this prediction (*SI Appendix*, sections 1.8–2.4). Both tests are presented as consistency checks, given their limited sample sizes.

### Perceived Intensity.

The dual-route architecture is formulated for recognition decisions and makes no specific predictions about subjective intensity. The phenomenological data we report here are therefore presented descriptively, with the architectural framework offered as one interpretive lens for a pattern that is striking in its own right. Perceived intensity patterns differed by etiology. In Moebius, a small interaction reflected a marginally wider ADFES–JeFEE gap, yet absolute ratings matched controls. In acquired palsy, participants down-rated JeFEE vividness (d ≈ 0.8) while judging ADFES normally, pinpointing postdevelopmental motor loss as the driver of this phenomenological shift. The contrast between cohorts is suggestive: postdevelopmental motor loss appears to alter the felt vividness of expressions whose recognition normally draws on sensorimotor reconstruction, while congenital absence of motor experience leaves absolute ratings aligned with controls, perhaps because lifelong experience calibrates subjective scaling against one’s own expressive landscape. We treat these readings as candidate interpretations rather than tests of the architecture. Full statistical outputs are in *SI Appendix*.

## Discussion

Across three studies, we exploited facial paralysis to ask under what conditions the sensorimotor system contributes to recognizing facial expressions, and how developmental history shapes those conditions.

The behavioral findings were consistent across cohorts. In both clinical groups, recognition accuracy scaled with residual motor capacity (SFGS) in a manner that depended jointly on stimulus ambiguity and etiology. For nonprototypical expressions the coupling appeared in both Moebius and acquired palsy; for prototypical expressions it appeared in Moebius alone, leading to a convergence for ambiguous stimuli and a dissociation for prototypical ones. Between-group differences in mean accuracy, by contrast, emerged only under the highest perceptual demand (the ultra-ambiguous morphs), not for the dynamic stimuli of the main studies. The pattern was robust to a broad set of alternative explanations, including autistic traits, alexithymia, depressive symptoms, perceived intensity, face-identity recognition, mid-level vision, age, education, and gender, and to lesion laterality in particular, which neither predicted accuracy within Moebius nor moderated the SFGS–accuracy coupling (*SI Appendix*, section 2.1.2).

Because the same stimulus sets and the same clinical grading metric yielded different coupling profiles across etiologies, the decisive advance is not simply that residual facial motor capacity predicts recognition, but that its predictive value depends jointly on perceptual demand and the developmental timing of motor loss.

Our results consolidate scattered findings into a single, testable account. Prior work showed that individuals with Moebius syndrome require stronger cues to detect morphed emotions (20) and argued that sensorimotor experience shapes facial-emotion processing (21), whereas studies of acquired palsy documented shifts in subjective appraisal for prototypical faces (16). We integrate these strands by demonstrating etiology-specific severity–performance relation.

**Fig. 6.**
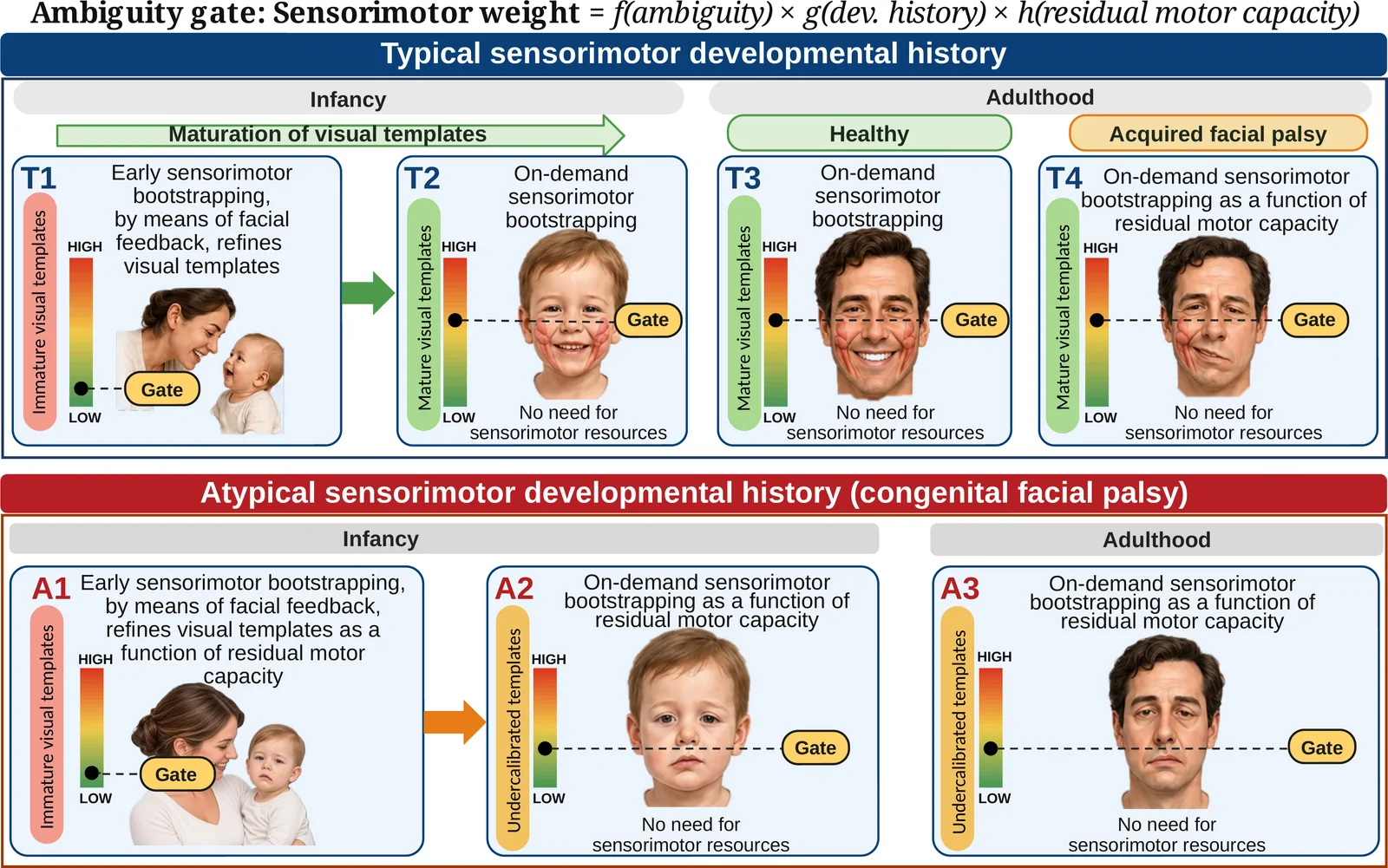
Proposed developmentally calibrated ambiguity-gated model. The schematic illustrates a mechanistic interpretation of the behavioral findings rather than a directly measured neural architecture, and separates the levels that future mechanistic work can test directly: developmental calibration, i.e., the slow refinement of visual templates across the developmental sequences (T1 through T3 for typical development, A1 through A3 for congenital palsy), online recognition (the trial-by-trial Gate within each panel), and candidate compensatory adaptation in congenital palsy (Lower panel). The architecture predicts the weight the recognition system places on sensorimotor information as a joint function of stimulus ambiguity, developmental history, and residual motor capacity (top equation). In each panel, the vertical bar on the left represents the maturation state of visual templates (labeled Immature, Mature, or Undercalibrated) and, correspondingly, the operative regime of perceptual ambiguity the system faces (red = high ambiguity/immature or undercalibrated templates; green = low ambiguity/mature templates). The marker labeled Gate indicates the threshold position on the ambiguity axis above which the recognition system draws on sensorimotor information; this position varies across panels as a function of developmental history and stimulus regime, while the underlying gating function (top equation) is preserved. Upper panel: Typical sensorimotor developmental history. During infancy (T1), visual templates are immature and perceptual ambiguity is correspondingly high; an early sensorimotor bootstrapping mechanism, driven by facial feedback from imitation (neonatal mimicry and caregiver mirroring), progressively refines visual templates toward canonical configurations. This maturation of visual templates carries the system into childhood (T2) and healthy adulthood (T3), where well-calibrated templates ground the recognition of prototypical expressions through the visual route alone, with on-demand sensorimotor bootstrapping available when stimulus ambiguity exceeds the gate’s threshold (the no need for sensorimotor resources label in T2–T4 and A2 to A3 refers to this subthreshold prototypical regime). When facial palsy is acquired after this calibration (T4), preonset templates remain intact and the operative regime continues to favor the visual route for prototypical recognition; the sensorimotor contribution drawn on for nonprototypical or otherwise ambiguous stimuli is now modulated by facial feedback as a function of residual motor capacity (indexed by the SFGS). Lower panel: Atypical sensorimotor developmental history (congenital facial palsy). In congenital palsy (A1), the same computational architecture for early sensorimotor bootstrapping remains available, but the facial feedback available to drive it is itself reduced—acting as a function of residual motor capacity. The maturation of visual templates is therefore incomplete, yielding undercalibrated visual templates in childhood (A2) and persisting into adulthood (A3). Undercalibrated templates leave residual perceptual uncertainty even for prototypical expressions, thereby broadening the stimulus range over which sensorimotor information becomes relevant to the recognition decision. The route itself, however, is shaped by the same congenital absence of motor experience that left templates undercalibrated: its functional integration with face-processing networks is reduced (Fig. 5), and prior work has suggested compensatory reorganization toward more ventral pathways (14). The severity–performance coupling observed in Moebius for both prototypical and nonprototypical expressions reflects the contribution of residual motor capacity to recognition across this widened stimulus range. Under the model, the gating function itself (top equation) is preserved across developmental histories; what differs is the representational and network substrate on which it operates—namely, undercalibrated visual templates and altered cross-route integration. Color bars represent perceptual ambiguity (red = high, green = low) and template maturation state. ChatGPT 5.6 Sol (OpenAI) was used under full human oversight solely to recreate the illustrative, non-data human figures in Fig. 6 as publication-quality graphical assets.

The behavioral results strongly support the framework we set out to test—ambiguity-gated and developmentally calibrated—while leaving the underlying mechanism to be tested directly. The pattern can be explained by two influences acting on different timescales (Fig. 6). Stimulus ambiguity governs when the visual route becomes insufficient: as an expression departs from stored templates, recognition comes to depend on the sensorimotor route, and residual motor capacity is coupled with performance—the coupling seen for nonprototypical expressions in both cohorts. Developmental motor experience governs the state of the templates themselves: where calibration was typical before onset, as in acquired palsy, clear expressions are resolved visually and severity is immaterial to them; where it was atypical from the start, as in Moebius, templates remain undercalibrated, so that even clear expressions would retain residual ambiguity and recognition of them, too, varies with severity.

That the Moebius pattern, and not the acquired one, shows severity effects for prototypical expressions points to the timing of motor experience rather than its mere presence: what distinguishes the two clinical cohorts is not whether facial movement is impaired but whether it was available early, while templates were presumably forming. This is what leads us to locate the developmental influence in a calibration of visual templates by early motor experience—and raises the question of how such calibration might occur. We consider early sensorimotor scaffolding a plausible route (Fig. 6, Upper panel). Prototypical-expression templates would be shaped through caregiver mirroring and transient neonatal mimicry (22–24), which provide proprioceptive input during a critical window of heightened plasticity—newborns imitate facial gestures within days and show μ-band desynchronization during facial observation by 3 mo (25, 26), and maternal mirroring predicts later μ-band suppression strength (27). This overt mimicry is transient, waning by the end of the first trimester (28). This early sensorimotor bootstrapping would consolidate mappings into stable visuo-sensorimotor representations, supporting later autonomous recognition while allowing the system to selectively draw on sensorimotor information (i.e., online sensorimotor bootstrapping) when expressions deviate from calibrated templates.

The gating principle at the core of this proposed architecture is not specific to faces. A convergent pattern was recently reported by Vannuscorps and Caramazza (29) in individuals born without upper limbs: action recognition was preserved for full-light displays but declined for point-light stimuli—precisely when visual input was degraded. Like our findings, this illustrates that visual pathways suffice when perceptual information is rich, whereas motor representations may become more informative under sensory uncertainty. This cross-domain correspondence suggests that ambiguity-gated recruitment of sensorimotor information is a general principle of adaptive embodiment, with plausible analogues in other channels through which emotion is read—vocal prosody, body posture, and their multimodal combination. More generally, the findings suggest a principle of adaptive perception: action-related information contributes most when sensory evidence is uncertain, while developmental experience calibrates how strongly that information is weighted.

This same architecture explains why aggregate embodiment effects often appear inconsistent. At the level of the field, the influential meta-analysis by Holland et al. (30) reported negligible associations between spontaneous facial mimicry and emotion recognition (r = −0.06, *P* = 0.54); within our framework, such apparent nulls are expected when studies are averaged across different regions of the ambiguity–severity continuum, where threshold-dependent contributions cancel out. The same logic resolves what might otherwise look like a paradox in our results: a robust severity–performance coupling within the patient cohorts alongside no mean difference between patients and controls. Under thresholded cooperation, this is no paradox. With prototypical dynamic stimuli, uncertainty is low, calibrated visual templates carry most of the load, and group means align; with ambiguous stimuli, rich kinematic information still lets many trials be solved visually, but within the patient cohorts the more ambiguous instances draw on the sensorimotor route, producing a severity–performance slope without shifting the mean. Secondary checks on independent data (19) were consistent with the interpretation that moving the task toward higher-demand regimes—by increasing stimulus ambiguity or transiently reducing facial feedback—made both severity coupling and group differences more likely (*SI Appendix*, sections 1.8 and 2.4).

Relative to matched controls, Moebius participants did not show attenuated μ-band ERD, yet exhibited weaker functional connectivity between sensorimotor and face-processing cortices (Fig. 5). This exploratory dissociation suggests that congenital facial palsy is associated with altered functional integration between sensorimotor and face-selective regions while leaving local sensorimotor responses preserved. This pattern is consistent with, but does not directly demonstrate, the idea that early sensorimotor bootstrapping primarily shapes the integration pathways. Future work using controlled manipulations of perceptual difficulty and higher-powered designs will clarify the conditions under which μ-ERD and network coupling become behaviorally diagnostic.

A methodological note bears mentioning: while our design exploits two independently validated corpora to operationalize the prototypicality contrast (ADFES for prototypical, JeFEE for nonprototypical configurations), a future study manipulating prototypicality within a single, fully controlled stimulus set, i.e., varying configural canonicality on the same actors and under matched recording conditions, would more directly isolate the prototypicality dimension and provide a stricter test of the architectural prediction. Such a parametric design would also bear directly on the graded character of the ambiguity gate: the architecture predicts a continuous reweighting between the two routes as a function of stimulus ambiguity, but the categorical (prototypical vs. nonprototypical) structure of our design supports this graded prediction only indirectly, through the within-cohort severity–performance coupling. A continuous ambiguity manipulation would allow direct testing of whether sensorimotor contribution scales monotonically with stimulus ambiguity, as the model predicts.

Future research will also need to explore the constructs that the paradigm operates on. Stimulus ambiguity, the perceptual property manipulated by the paradigm, is related but not identical to perceiver-side uncertainty, the epistemic state that the gate actually operates on. A nonprototypical stimulus is objectively ambiguous, but it only produces effective uncertainty relative to the perceiver’s calibrated templates. The Moebius pattern is a useful example in this regard. Severity-performance coupling for nominally prototypical expressions, according to our account, reflects an increase in effective uncertainty rather than any change in the objective ambiguity of the stimuli themselves. Sensorimotor bootstrapping, in this view, is fundamentally a process of uncertainty calibration. It tunes the perceiver’s templates to handle the typical variability of expressive input, drawing on sensorimotor support where those templates fall short. To empirically disentangle stimulus ambiguity from perceiver-side uncertainty, researchers can use confidence ratings, uncertainty-decoding paradigms, or independently manipulate template calibration and stimulus prototypicality.

Another important area of research is the role of context. In ecological emotion perception, faces are rarely processed in isolation. Situational cues, social setting, prior expectations, vocal prosody, and body posture all contribute to disambiguating any given expression. Our paradigm, like most laboratory studies of emotion recognition, deliberately removes these contextual factors to isolate the stimulus contribution. While this approach provides a controlled picture, it is necessarily incomplete. The question arises whether contextual information attenuates the gate (lowering the threshold at which sensorimotor support becomes necessary), substitutes for it (reducing reliance on sensorimotor reconstruction altogether), or interacts with developmental calibration as a possible compensatory mechanism in congenital motor experience deficits. The present design cannot address these questions. In addition to developmental studies of how uncertainty calibration unfolds across infancy and beyond, this ecological dimension positions the architecture as a candidate framework for understanding uncertainty representation across developmentally calibrated systems.

Taken together, our findings support a thresholded, developmentally calibrated framework in which stimulus ambiguity and early motor experience jointly shape when sensorimotor information becomes behaviorally relevant. We present this as a strong behavioral foundation rather than a direct measurement of the underlying mechanisms. A critical next step is therefore to determine the distinct contributions of efferent motor commands, peripheral somatosensory feedback, and central sensorimotor representations—components that the present natural experiment cannot disentangle. By specifying where the behavioral signature of sensorimotor contribution should appear—across levels of ambiguity, developmental timing, and residual motor capacity—the framework identifies the experiments that would test these mechanisms directly: parametric manipulation of perceptual ambiguity; selective perturbations designed to differentially alter facial motor output and afferent somatosensory feedback, combined with concurrent central and peripheral sensorimotor measures; and—critically for the developmental claim—tracking how the coupling between motor capacity and recognition emerges across early development, when template calibration is hypothesized to occur. Clinically, the framework generates the testable prediction that developmental history—not impairment severity alone—will moderate the benefit of interventions that restore or substitute facial sensorimotor feedback. The framework thereby reframes the long-standing vision-vs.-embodiment debate as a more sharply defined program of work.

## Materials and Methods

A total of 185 participants were recruited across three studies. Study 1: 117 neurotypical adults (104 female; mean age = 21.69 ± 5.72 y). Study 2: 15 individuals with Moebius syndrome (7 male, 8 female; mean age = 35.27 ± 15.50 y) and 15 control participants matched to the Moebius cohort on age (±2 y), sex, and years of formal education (±2 y), representing approximately 3% of all diagnosed cases in Italy. Study 3: 19 individuals with acquired facial palsy (15 female, 4 male; mean age = 45.79 ± 13.55 y; etiologies: iatrogenic n = 8, idiopathic/Bell’s palsy n = 4, viral n = 3, vascular n = 3, inflammatory n = 1), recruited over 2 y from three specialized centers, and 19 control participants matched to the acquired palsy cohort on age (±2 y), sex, and years of formal education (±2 y). Notably, this constitutes the largest neurophysiologically characterized Moebius sample to date, extending earlier behavioral and autonomic work (2, 13, 14, 19, 20, 21, 31, 32). The full study protocol was approved by the Ethics Committee of the University of Padova (Protocol No. 4842). All participants provided written informed consent prior to participation, in accordance with the Declaration of Helsinki.

Palsy severity was quantified with the SFGS (33, 34), a validated composite of resting symmetry, voluntary excursion, and synkinesis (range 0 to 100), administered by a single senior physiotherapist (~15 y experience), blinded to hypotheses and to the participant’s performance on the experimental task. The SFGS was administered only to clinical participants. The instrument is designed and normed as a measure of facial-nerve impairment relative to unimpaired function; for participants without facial palsy, it, by construction, yields a ceiling score and does not yield interpretable individual variability. The assessment battery included alexithymia (TAS-20; 35), autistic traits (AQ-50; 36), face-identity processing (Oxford Face Matching Test; 37), and mid-level visual perception (L-POST; 38) for Study 2, and depression (MADRS; 39) for Study 3 (*SI Appendix*). Control variables were selected on the basis of population-specific confound profiles documented in the clinical literature for each cohort.

Participants viewed validated dynamic expressions from ADFES (prototypical; 17) and JeFEE (nonprototypical; 18): 28 clips per set, 4 actors (2 female), and the same six basic expressions plus neutral expressions for both datasets (6 to 11 s). Each clip was presented twice across the experimental session, yielding 8 trials per emotion category per stimulus set. ADFES comprises dynamic videos of trained actors instructed to produce maximally clear, FACS-validated configurations of the basic emotions, yielding canonical, high-prototypicality expressions; JeFEE comprises dynamic expressions of the same six categories produced by nonactor expressers without FACS-based instructions, yielding configurally noncanonical, lower-prototypicality expressions. The two corpora index categorically distinct properties rather than two intensities of the same property: in the original normative work, the JeFEE expressions were shown to recruit muscular configurations that differ qualitatively from those of low-intensity stereotypical expressions, and were rated by independent observers as more representative of expressions encountered in everyday social interaction (18). This dissociation between actor-based, FACS-instructed canonical productions, and nonactor spontaneous productions is what justifies treating ADFES and JeFEE as endpoints of a prototypicality continuum rather than as graded versions of the same expressive template.

Responses were collected via the Geneva Emotion Wheel (GEW 1.0; 40). On each trial, a fixation cross appeared at the center of the screen for 500 ms, followed by a facial expression video clip (6,000 to 10,000 ms). The GEW was then presented—a circular arrangement of 16 emotion labels organized by valence and control/power, plus a center representing neutral and peripheral arcs encoding intensity—and participants used the mouse to select the position best matching both the perceived emotion and its intensity, within a 20-s response window. Responses were scored as correct when the response angle fell within the angular sector corresponding to the target basic-emotion category (full procedure in *SI Appendix*, section 1.6). Study 2 was laboratory-based (PsychoPy v2020.1.3); Studies 1 and 3 were online (Gorilla Experiment Builder; 41). Full stimulus and scoring details are in *SI Appendix*.

High-density EEG was recorded from 128 channels at 500 Hz during facial expression viewing in the Moebius cohort and matched controls. We analyzed two markers: i) μ-band (8 to 13 Hz) event-related desynchronization (ERD) over sensorimotor and visual cortices, and ii) source-level functional connectivity between sensorimotor cortices and face-processing regions (fusiform gyrus, STS), using corrected imaginary phase-locking values. Full preprocessing and analysis details are in *SI Appendix*.

Because cohort size was fixed by the rarity of these conditions, we specified an a priori hierarchical analytic strategy that distinguished confirmatory behavioral inference from planned exploratory neural analyses and additional exploratory tests. Confirmatory behavioral analyses comprised: i) a generalized linear mixed model (binomial, logit) contrasting JeFEE and ADFES accuracy; and ii) within each clinical cohort, correlations between SFGS scores and recognition accuracy, with Benjamini–Hochberg FDR control. The neural analyses—μ-band ERD and sensorimotor–visual connectivity contrasts between Moebius participants and controls—were also specified a priori, but were treated as exploratory because the Moebius sample was not powered for condition-specific neural inference. Additional exploratory analyses included Group × Expression interactions, mediation models, leave-one-emotion-out robustness checks, and ancillary regressions. Where appropriate, estimates were accompanied by effect sizes, 95% BCa bootstrap CI, Bayes factors, leave-one-out cross-validation, and sensitivity analyses. Power planning for the primary clinical correlations used a target effect of r = 0.60, 80% power, and one-tailed α = 0.05. The confirmatory behavioral and planned neural analyses were specified a priori but not preregistered; all remaining analyses were exploratory. The use of generative artificial intelligence was classified according to the Generative Artificial Intelligence Delegation Taxonomy (42).

## Supplementary Material

Appendix 01 (PDF)

## Data Availability

Behavioral datasets, EEG data, R analysis scripts, and experimental protocols have been deposited in the Open Science Framework (OSF) (43) [Facial palsy reveals the sensorimotor contribution to facial-emotion recognition].
