# Supplementary material for: Facial palsy reveals the sensorimotor contribution to facial-emotion recognition
Source: Proc Natl Acad Sci U S A. 2026 Sep 11;123(37):e2608511123. doi: 10.1073/pnas.2608511123 (PMC13578938; doi:10.1073/pnas.2608511123)
Supplement: Supplementary file 1 [file pnas.2608511123.sapp.pdf]

SI Appendix

Facial palsy reveals the sensorimotor contribution to facial-emotion recognition

Paola Sessa[a,b], Arianna Schiano Lomoriello[a,b,1,2], Thomas Quettier[a,b,1,3], Antonio Maffei[a,b], Sara Costa[c], Marta Nichele[d], & Pier Francesco Ferrari[c,e]

[a]Department of Developmental Psychology and Socialisation, University of Padova, Padova 35131, Italy; [b]Padova Neuroscience Center, Department of Developmental Psychology and Socialisation, University of Padova, Padova 35129, Italy; [c]Department of Medicine and Surgery, University of Parma, Parma 43126, Italy; [d]Independent Physiotherapist, Padova 35143, Italy; [e]Social Neuroscience and Comparative Development, Social Neuroscience and Comparative Development, Institut des Sciences Cognitives Marc Jeannerod, CNRS/Université Claude Bernard Lyon 1, Bron Cedex 69675, France

FOOTNOTES:

1 A.S.L. and T.Q. contributed equally to this work.

2 To whom correspondence may be addressed. Email: arianna.schianolomoriello@unipd.it.

3 Present address: Department of Medical and Surgical Sciences, Magna Graecia University of Catanzaro, Catanzaro 88100, Italy

Classification: Biological Sciences – Psychological and Cognitive Sciences

Keywords: embodied cognition | facial paralysis | emotion recognition | sensorimotor calibration | Moebius syndrome

Table of Contents

1. **Supplementary Methods**

    1.1 Theoretical Rationale and Effect-Size Justification

    1.2 Psychometric & Clinical Instruments (Studies 2 & 3)

        1.2.1 Sunnybrook Facial Grading System (SFGS)

        1.2.2 Toronto Alexithymia Scale (TAS-20)

        1.2.3 Autism Spectrum Quotient (AQ-50)

        1.2.4 Montgomery–Åsberg Depression Rating Scale (MADRS)

    1.3 Additional Behavioral & Visual Tasks (Study 2)

        1.3.1 Oxford Face Matching Test (OFMT)

        1.3.2 Leuven Perceptual Organization Screening Test (L-POST)

    1.4 Stimuli & Apparatus

        1.4.1 Video Stimuli (ADFES & JeFEE)

        1.4.2 Geneva Emotion Wheel (GEW)

        1.4.3 Hardware & Software Specifications

    1.5 Statistical Analysis Plan

    1.6 Behavioral Analysis

        1.6.1 Recognition Accuracy & Intensity in Healthy Participants (Study 1)

        1.6.2 Group Comparisons for Accuracy & Intensity (Studies 2 & 3)

        1.6.3 Reference Sample Comparisons (Studies 2 & 3)

        1.6.4 Correlation & Cross-Cohort Analyses (Studies 2 & 3)

    1.7 Detailed EEG Methods & Analysis (Study 2)

        1.7.1 Acquisition & Pre-processing

        1.7.2 Time-Frequency Analysis

        1.7.3 Functional Connectivity

1.8 Morphed Intensity-Rating Paradigm (Reanalysis; ref. 27)

2. **Supplementary Results**

2.1 Study 2 (Congenital Facial Palsy)

2.1.1 Behavioral Results

2.1.2 Mediation Analyses

2.2 Study 3 (Acquired Facial Palsy)

2.2.1 Behavioral Results

2.2.2 Mediation Analyses

2.3 Summary of Patient–Control Behavioral Comparisons

2.4 Intensity-Rating Task (ref. 27): Between-Group Results

2.5 Intensity-Rating Task (ref. 27): Severity–Performance Correlations in the Moebius Group

2.6 Robustness of the aggregate recognition score across emotion categories

3. **Supplementary Tables**

Supplementary Table 1. Demographic & Clinical Characteristics — Study 2

Supplementary Table 2. Demographic & Clinical Characteristics — Study 3

Supplementary Table 3. Leave-one-emotion-out analysis of the aggregate recognition score — Study 2 and 3

4. **Supplementary Figures**

Supplementary Fig. 1. Geneva Emotion Wheel (Italian Translation)

Supplementary Fig. 2. Accuracy & Perceived Intensity — Group Comparisons (Study 2)

Supplementary Fig. 3. SFGS–accuracy relationship by facial lateralisation (Study 2)

Supplementary Fig. 4. Accuracy and Perceived Intensity, Group Comparisons (Study 3)

Supplementary Fig. 5. Morphed Intensity-Rating group comparison (27)

Supplementary Fig. 6. SFGS–emotion recognition correlations in Moebius syndrome

Supplementary Fig. 7. SFGS–emotion recognition relationship across etiologies

5. **Supplementary References**

1. Supplementary Methods

1.1 Theoretical Rationale and Effect-Size Justification

Because no prior effect sizes were available for motor-recognition correlations in these specific clinical populations, we adopted a theory-driven approach to determine our target effect size. We designated r = 0.60—explaining 36% of the variance—as the minimum effect of theoretical and clinical interest for our primary hypothesis that facial-motor proficiency predicts emotion-recognition accuracy. This threshold was chosen based on several considerations: (1) if sensorimotor input makes a substantive contribution to facial expression processing, the effect should be large enough to be clinically meaningful, (2) correlations below r = 0.60 would suggest that motor function accounts for less than 36% of recognition variance, potentially indicating that alternative mechanisms (e.g., purely visual processing) dominate, and (3) this effect size represents a large correlation by conventional standards while remaining achievable given the direct theoretical relationship between motor capacity and the contribution of sensorimotor information to recognition.

1.2 Psychometric & Clinical Instruments (Study 2 and Study 3)

Moebius syndrome is characterized not only by congenital facial palsy but also by deficits in eye movement production resulting from VI cranial nerve involvement. Previous research has documented impairments in eye movement control and mid-level perceptual organization among individuals with Moebius syndrome. These deficits have been identified as

potential confounding factors in studies investigating facial expression recognition, raising the possibility that observed impairments could be mistakenly attributed to general perceptual deficits rather than the absence of facial mimicry. To control for these variables, we administered a comprehensive battery of low- and mid-level visual processing tasks to determine whether any observed differences in facial expression perception could be attributed to general visual deficits rather than impairments specifically affecting emotional processing.

1.2.1 Sunnybrook Facial Grading System (SFGS) (Study 2 and Study 3)

Severity of facial paralysis in both clinical cohorts was assessed using the Sunnybrook Facial Grading System (SFGS; 1, 2), a standardized clinician-rated instrument providing a comprehensive index of facial nerve function. The SFGS evaluates three components—resting symmetry, voluntary muscle movement, and synkinesis—across five standardized facial actions (forehead wrinkle, gentle eye closure, open-mouth smile, snarl, and lip pucker). Each movement is scored from 1 to 5, with higher values reflecting better motor function, and total scores range from 0 to 100 (lower scores indicating more severe paralysis). All evaluations were performed by the same physiotherapist, an expert in facial paralysis, who remained blinded to all other aspects of the study and completed assessments independently of the data-collection sessions. SFGS scores were merged with the rest of the study data only after all evaluations had been finalized.

1.2.2 Toronto Alexithymia Scale (TAS) (Study 2)

Participants in Study 2 completed the Toronto Alexithymia Scale-20 (TAS-20; 3), a validated self-report questionnaire measuring difficulties in identifying and describing feelings and a tendency toward externally oriented thinking. We included this measure because

alexithymia has been shown to influence emotion-recognition performance in both behavioral and neurocognitive tasks, and may therefore contribute to group-level differences.

1.2.3 Autism Spectrum Quotient (AQ-50) (Study 2)

Autistic traits were assessed using the Autism Spectrum Quotient—50 item version (AQ-50; 4), a widely used self-report questionnaire designed to measure autistic characteristics in the general population. The AQ includes five subscales—Social Skills, Attention Switching, Attention to Detail, Communication, and Imagination—each comprising 10 items rated on a 4-point Likert scale. Total scores range from 0 to 50, with higher values indicating stronger autistic traits; scores ≥ 32 are typically considered indicative of clinically significant levels.

1.2.4 Montgomery–Åsberg Depression Rating Scale (MADRS) (Study 3)

In Study 3 (acquired facial paralysis), we administered the MADRS (5) alongside the SFGS to account for potential mood-related confounds that may accompany peripheral facial-nerve injury, although the prevalence of clinically relevant symptoms in this population remains uncertain. The MADRS is a 10-item semi-structured clinician-administered interview covering core affective and vegetative domains (e.g., apparent/reported sadness, lassitude, anhedonia, concentration problems, and sleep or appetite changes). Each item is scored from 0 to 6, yielding a total score of 0–60, with higher values indicating more severe depressive symptoms. Interviews were conducted by a trained clinical psychologist following standardized administration guidelines, ensuring that ratings were anchored to defined symptom thresholds and referred to the previous seven days.

1.3 Additional Behavioral & Visual Tasks (Study 2)

1.3.1 Oxford Face Matching Test (OFMT)

Face identity recognition abilities were assessed using the Oxford Face Matching Test (OFMT) (6), implemented in the Gorilla experiment builder. This measure was included to determine whether group differences in the emotion-intensity judgment task could stem from variability in high-level visual processing abilities rather than other cognitive factors. On each trial, participants viewed two faces presented side by side for 2,000 ms and indicated whether they depicted the same individual or different people. We adopted a fixed stimulus presentation time to minimize response bias; after stimulus offset, participants responded at their own pace without time limit.

Stimuli consisted of grayscale frontal-view portraits of male and female Caucasian individuals (18 to 70 years), displayed against a white background, with hair, jawline, and ears fully visible. Participants completed 200 trials (four blocks of 50), presented in randomized order. Accuracy was scored as the number of correct responses (0–200).

The original OFMT uses a 1,600-ms presentation time, but we extended this to 2,000 ms because stimuli were displayed in pairs and individuals with Moebius syndrome have congenital cranial nerve VI palsy affecting ocular motility. This adjustment ensured that performance differences reflected face-processing ability rather than oculomotor limitations that might hinder scanning and comparing the face pairs.

1.3.2 Leuven Perceptual Organization Screening Test (L-POST)

Each L-POST subtest (7) consisted of five trials in which participants viewed a target stimulus alongside three alternatives and selected the option that best matched the target based on the relevant perceptual principle. Completion time ranged from 20 to 45 minutes depending

on individual pace. Performance was automatically scored, enabling quantitative comparison with age-matched normative data.

1.4 Stimuli & Apparatus (Studies 1-3)

1.4.1 Video Stimuli (ADFES & JeFEE)

The experimental stimuli consisted of dynamic facial expressions portraying the six basic emotions (anger, disgust, fear, happiness, sadness, and surprise) as well as neutral expressions. Eight actors (four female) were selected from two validated datasets: (1) the Amsterdam Dynamic Facial Expression Set (ADFES; 8; actors F01, F03, M02, M03), which provides prototypical, high-intensity emotional expressions; and (2) the Jerusalem Facial Expressions of Emotion (JeFEE) dataset (9; actors JF1, JF2, JM1, JM2), which features subtler, less intense, and non-prototypical expressions.

ADFES clips lasted 6–6.5 seconds: actors held a neutral expression for 0.5 seconds before transitioning to an emotional apex lasting 5–6 seconds. These expressions were developed using the Facial Action Coding System (FACS) to ensure high prototypicality and intensity of facial muscle activation. In contrast, JeFEE clips were 10–11 seconds in duration, with expressions unfolding gradually from neutral to full expression. Unlike ADFES, where actors engaged specific muscle configurations to reach standardized expressions, JeFEE actors produced more naturalistic movements without FACS-based constraints.

All videos from both datasets were converted to MP4 format and resized to a uniform 700 × 700 px resolution using VideoProc Converter (Version 4.1; videoproc.com).

1.4.2 Geneva Emotion Wheel (GEW)

Participant responses were collected using the Geneva Emotion Wheel (GEW 1.0; 10, 11). GEW 1.0 comprises 16 discrete emotion terms arranged in a circular layout defined by two underlying dimensions: Valence (pleasant–unpleasant) and Control/Power (high–low). Emotions occupy four quadrants according to their position on these dimensions. The center of the wheel represents a neutral state, and emotional intensity increases progressively from the center toward the periphery (Supplementary Fig. 1).

Consistent with Russell's circumplex model of affect (12), the GEW represents emotions both discretely and continuously: emotions with similar valence and control characteristics form adjacent "rays" along a continuum, whereas emotions with contrasting properties appear in diametrically opposite positions. This structure enabled a systematic assessment of both emotion-intensity judgments and categorical emotion recognition for each video stimulus. All emotion terms were translated into Italian for administration.

We specifically employed GEW version 1.0 (16 emotions + neutral; four intensity levels) for three main reasons: 1) Its graphic design is user-friendly, allowing participants to grasp the underlying principle quickly. 2) It avoids the "ceiling effect," as participants are required to identify the correct emotion amongst various distractors. 3) It encompasses the basic emotion of "surprise," which is not included in the latest version (3.0).

1.4.3 Hardware & Software Specifications

The laboratory experiment (Study 2) was administered using PsychoPy v2020.1.3. Visual stimuli were presented on an Asus ROG PG248Q 24″ monitor (1920 × 1080 pixels, 144 Hz; brightness: 100, contrast: 50), driven by an NVIDIA GeForce GTX 1050 Ti 4GB graphics card. At a viewing distance of 70 cm, stimuli subtended approximately 18° of visual angle. Participants were tested individually in a dimly lit, acoustically controlled room. A chin rest

was not used to preserve a more naturalistic posture, although participants were instructed to maintain a stable head position throughout.

Studies 1 and 3 (administered online) were built and hosted using the Gorilla Experiment Builder (www.gorilla.sc; 13), which allowed us to replicate the PsychoPy implementation. To ensure consistent visual presentation, access was restricted to desktop and laptop devices; tablets and smartphones were blocked. Across participants, screen resolutions ranged from 1093 to 2560 pixels in width and 615 to 1440 pixels in height.

Before beginning the task, participants completed a visual-scaling calibration to standardize the physical display size. They were instructed to place a standard credit card against their screen and adjust an on-screen slider until the on-screen reference matched the physical card. This procedure ensured that the stimuli were displayed at a comparable physical size across devices, approximating the presentation parameters of the laboratory setting.

1.5 Statistical Analysis Plan

To address the limited sample sizes characteristic of rare-disease cohorts while maintaining transparent inferential standards, we implemented a hierarchical analytic framework distinguishing confirmatory behavioural analyses, planned exploratory neural analyses, and additional exploratory or robustness analyses, as detailed in the main manuscript.

1.6 Behavioral Analysis

All behavioral statistical analyses were performed using R (version 4.3.1). We employed both frequentist and Bayesian statistical approaches to provide a comprehensive and convergent evaluation of the behavioral data. We first established performance benchmarks for facial expression recognition and perceived intensity using a large sample of healthy participants (Study 1, n = 117). This dataset served as the reference for subsequent clinical comparisons. To compute accuracy based on participants' responses using the GEW, we first transformed Cartesian coordinates (x_{ij}, y_{ij}) into polar coordinates (r_{ij}, θ_{ij}) for each trial, as shown in the Equation:

$$\theta_{ij} = tan^{-1}\left(\frac{x_{ij}}{y_{ij}}\right)$$

This transformation yielded the response angle for each trial. Since each emotion category occupies a defined angular sector on the GEW, we assessed performance using a position-independent accuracy metric: a trial was considered accurate if the response angle θ_{ij} fell within the angular boundaries of the target emotion's sector (i.e., the corresponding "slice" of the GEW). Neutral expressions occupied the center of the GEW without angular boundaries, preventing the accuracy calculation. These trials (n=16 per participant) were excluded from accuracy analyses but retained for intensity analyses, where radial distance from center provided a valid metric. The emotion intensity is expressed as the difference from the center of the GEW. Values close or far from the center represent, respectively, neutral and high facial expression intensity. We calculated the intensity for each trial as the Euclidean distance between the center and the location where the button was pressed. Given that the GEW has been centered (i.e., the center has coordinates ($x = 0, y = 0$), the distance from the center is calculated as in the Equation:

$$I_{ij} = \sqrt{x^2 + y^2}$$

1.6.1 Recognition Accuracy and Intensity in Healthy Participants (Study 1)

Recognition accuracy was assessed for both ADFES and JeFEE facial expressions. For each participant, mean accuracy was calculated separately for each expression type. Descriptive statistics were computed for each condition, including the mean and standard deviation, providing a clear picture of typical performance and its variability. To formally assess the effect of prototypicality, we fit a generalized linear mixed model (GLMM) with prototypicality (ADFES vs. JeFEE) as a within-subjects factor. Accuracy was analysed as grouped binomial data (proportion correct with the corresponding number of trials specified as weights), allowing the model to account for the binomial nature of the outcome while incorporating participant-level random effects. The GLMM provided estimates of effect size (partial eta squared; Cohen's d for repeated measures) and enabled direct interpretation of within-subject contrasts.

In parallel, we conducted Bayesian ANOVAs, reporting Bayes factors (BF_{10}) to quantify the strength of evidence supporting observed effects.

Intensity ratings were analyzed using a repeated-measures ANOVA, with descriptive statistics computed for each condition.

Together, these analyses yielded reference distributions for accuracy and intensity that served as normative benchmarks. These distributions were subsequently used to determine the range of typical performance and to contextualize deviations observed in patient and control groups in Studies 2 and 3.

1.6.2 Group Comparisons for Accuracy and Intensity (Studies 2 and 3)

For both the Moebius syndrome cohort (Study 2) and the acquired facial palsy cohort (Study 3), recognition accuracy was analysed using generalized linear mixed models, whereas perceived-intensity ratings were analysed using mixed-design ANOVAs with expression type as a within-participant factor and group as a between-participant factor. Each model included

expression type (ADFES vs. JeFEE) and group (Moebius syndrome vs. control or acquired facial palsy vs. control) factors. The analysis framework was designed to test three key effects: 1) Main effect of expression type (ADFES vs. JeFEE), examining whether prototypical and non-prototypical, ambiguous expressions were processed differently; 2) Main effect of group (patient vs. control), assessing overall performance differences between groups; 3) Expression × group interaction, testing whether any prototypicality effects were modulated by group membership. We calculated descriptive statistics for each condition and group (mean, standard deviation). Effect sizes were quantified using partial eta squared (η_p^2) for ANOVA effects and Cohen's d for specific contrasts. The magnitude of η_p^2 effects was interpreted according to standard benchmarks: small (0.01), medium (0.06), and large (0.14). Where interactions were significant, follow-up contrasts were conducted with p-values adjusted for multiple comparisons using the false discovery rate (FDR) correction.

This analytical approach allowed us to: 1) Quantify the relative impact of expression type and group membership on performance; 2) Assess whether any group differences were specific to specific expression types; 3) Compare the magnitude of prototypicality effects between patient and control groups; 4) Evaluate both overall performance differences and specific pattern differences between groups.

1.6.3 Reference Sample Comparisons (Study 2 and Study 3)

While our primary analyses revealed robust motor-recognition correlations within patient groups, exploratory between-group comparisons painted a more nuanced picture. Despite the limited statistical power to detect group differences, these analyses provide important context for understanding how facial paralysis affects emotion recognition at the population level.

To evaluate whether patients and controls from Studies 2 and 3 performed similarly to the reference sample from Study 1 (n = 117), we conducted a comprehensive set of statistical comparisons for both accuracy and intensity ratings. For each group (Moebius syndrome, acquired facial palsy, and their respective controls), we performed one-sample t-tests against the reference cohort means, separately for ADFES and JeFEE stimuli. Effect sizes were calculated as Cohen's d, with negative values indicating performance decrements relative to norms. To complement the frequentist analysis, we computed Bayes factors (BF_{10}) using a default Cauchy prior (scale = 0.707) to quantify the evidence for performance differences from the reference sample. The magnitude of any observed decrements was then compared between patient and control groups using Fisher's r-to-z transformation, with the resulting z-scores tested against the null hypothesis of no between-group differences in effect size.

1.6.4 Correlation and Cross-Cohort Analyses (Study 2 and Study 3)

To examine the relationship between facial motor function and emotion recognition, we implemented a multi-level correlation analysis framework. Within both Moebius syndrome and acquired facial palsy cohorts, we computed Pearson correlation coefficients between SFGS scores and two dependent measures: recognition accuracy for ADFES and recognition accuracy for JeFEE. For each correlation, we calculated exact p-values, effect sizes (r), and Bayes factors (BF_{10}) using a default Cauchy prior (scale = 0.707). The stability of these correlations was assessed through non-parametric bootstrap resampling (10,000 iterations), which generated 95% confidence intervals and empirical sampling distributions. To validate these relationships, we conducted ElasticNet regression analyses, computing R^2 values and standardized coefficients. To test the generalizability of these associations across etiologies, we implemented three complementary analyses. First, correlation coefficients were transformed using Fisher's r-to-z method to normalize their sampling distributions. Second, we conducted a joint

probability analysis combining p-values from both cohorts using Fisher's method. Third, we compared the overlap of bootstrap-derived confidence intervals between groups. To control for severity differences between cohorts, we conducted a planned matched-cohort analysis. Participants were selected based on overlapping SFGS scores (range: 19-76) to create matched subgroups from both populations. Within these severity-matched groups, we computed correlations between motor function and recognition accuracy, with significance tested using both frequentist (p-values) and Bayesian (BF_{10}) approaches. Potential mediating factors were examined through parallel mediation analyses. For Moebius syndrome, we tested autistic traits (Autism Quotient) and alexithymia (Toronto Alexithymia Scale) as mediators. For acquired palsy, we examined depression symptoms (Montgomery-Åsberg Depression Rating Scale). Each mediation model estimated total effects, direct effects, and indirect effects through proposed mediators, with standardized coefficients (β) and p-values reported for all pathways. For descriptive purposes, LOESS-smoothed curves (span = 0.75) with pointwise 95% confidence bands were plotted for the two Moebius stimulus sets and for acquired-palsy JeFEE performance (Supplementary Fig. 7).

1.7 Detailed EEG Methods and Analysis (Study 2)

1.7.1 Acquisition & Pre-processing

Electroencephalography (EEG) was recorded from 128 channels using an R-Net sponge electrode cap connected to a BrainAmp DC amplifier. Data were sampled at 500 Hz with the vertex as the online reference. Electrode impedance was maintained below 50 kΩ.

Data preprocessing was performed in MATLAB using functions from EEGLab (v.2021) and Brainstorm, and consisted in the following steps: i) removal of line noise artifact using the

ZapLine algorithm (14), ii) high-pass filter the data at 0.1 Hz, iii) automatic detection and removal of bad channels using the clean_rawdata plugin, defining a channel as bad if flat for more than 5 seconds and/or if displaying a correlation with neighboring channels less than 0.8, iv) segmenting the data into epochs ranging from −1 to 6 s relative to stimulus onset for ADFES trials and from −1 to 10 s for JeFEE trials; v) applying independent component analysis and semi-automatically identifying artifact-related components using ICLabel (15); vi) detecting and removing epochs containing residual artifacts using an amplitude threshold of ±100 μV; and vii) interpolating removed channels using spherical splines.

1.7.2 Time-Frequency Analysis

Time-varying changes in power spectral density were quantified using the short-time Fourier transform (STFT) to extract the power in frequencies ranging from 2 to 30 Hz. Analysis was then restricted to the alpha/mu band (8-13 Hz), an oscillatory range typically associated with sensorimotor and action simulation processes. Baseline correction was applied by subtracting the mean spectral power in the pre-stimulus interval (-1 to 0 s). Event-related synchronization/desynchronization (ERS/ERD) was then computed as

$$ERS/ERD = \frac{E - \mu}{\mu} * 100$$

, where E represents the power density during the event-related period, and μ corresponds to the mean power density during the baseline.

Two regions of interest (ROIs) were selected for analysis. The central region, associated with μ rhythm suppression, included the following electrodes: C1, C3, C5, Cz, FCC3h, FCC5h, FC1, FC3, FC5, CCP5h, CCP3h, CCP1h, CP5, CP3, and CP1 (left hemisphere); and C2, C4, C6, FCC2h, FCC4h, FC2, FC4, FC6, CCP6h, CCP4h, CCP2h, CP6, CP4, CP2, and CPz (right hemisphere). The posterior region, serving as a control measure to verify that desynchronization

in central electrodes was not simply a reflection of global cortical suppression, comprised electrodes in the occipital and parieto-occipital areas: PPO3h, PPO5h, PO3, PO7, PPO9h, O1, O2, Oz, OI1h, I1, Iz, POz, and POO1 (left hemisphere); and PPO4h, PPO6h, PO4, PO8, PPO10h, OI2h, I2, and POO2 (right hemisphere). Posterior alpha suppression was employed as a control measure to ensure that μ-rhythm desynchronization was specific to central electrodes and did not reflect global cortical suppression, given that posterior alpha oscillations are primarily associated with visual attention rather than sensorimotor processing (16, 17). Anterior μ-ERD, by contrast, has long been considered a marker of sensorimotor engagement during action observation, including facial movements, yet previous work reports mixed links to performance: stronger right-hemisphere μ-ERD correlates with empathic accuracy—a construct that overlaps with but is not isomorphic to basic emotion labelling (18)—whereas earlier—but not larger—μ-ERD peaks differentiate high- from low-accuracy observers (19). Overall, this previous evidence together with our own data might suggest that the functional benefit of μ-ERD is determined by spatiotemporal dynamics—timing, lateralization and embedding within the visuo-sensorimotor network—rather than by raw amplitude only.

1.7.3 Functional Connectivity

Source-level connectivity was quantified by first projecting the scalp-level data to source space using the weighted Minimum Norm Estimation (wMNE) as inverse solution, then subsampling the estimated solution to the 148 cortical parcels of Destrieux et al. (20), averaging the activity of all the vertices included in each parcel. Functional connectivity in the alpha/mu band (8-13 Hz) was estimated using the phase locking value, a widely used statistic that quantifies the degree of phase synchronization in a given frequency band (21–23). Specifically, we used the corrected imaginary part of PLV (ciPLV), which for each pair of signals is defined as the imaginary part of the phase difference between the two signals (24), thus discarding zero-

lag interactions, and providing an estimation more robust to volume conduction and/or source leakage. For the goal of the present study, we estimated connectivity strength between regions comprised in the core system (CS) of the face processing network and the primary and secondary motor and somatosensory cortices comprised in the sensorimotor system (SMS) (22, 25, 26), defining the connectivity strength between the two systems as the sum of the connectivity matrix. Additionally, we computed connectivity between a specific face-processing region in the core system—the superior temporal sulcus (STS)—and the regions comprising the sensorimotor system.

1.8 Morphed Intensity-Rating Paradigm (Reanalysis; ref. 27)

We reanalysed behavioural data from the intensity-rating experiment described by Schiano Lomoriello et al. (27), including eight Moebius participants (whose Sunnybrook Facial Grading System (SFGS) scores were collected in the present study) and neurotypical controls (n = 11). In addition, we analysed data from a facial mask condition (n = 11), which are reported in the present SI Appendix (Section 2.4). In this condition, neurotypical participants wore facial hardening masks to transiently dampen facial feedback—a manipulation shown to impair visual discrimination of morphed facial expressions in neurotypical adults (28).

Participants rated the perceived intensity (1–10 scale) of morphed facial expressions spanning from neutral to full emotion across six categories (anger, disgust, fear, happiness, sadness, and surprise). Each face was rated for all six emotions in separate blocks, creating high perceptual ambiguity and requiring fine-grained discrimination of emotional intensity.

Full methodological details, stimulus construction, and timing parameters are available in Schiano Lomoriello et al. (27). Here, we focused on three behavioural metrics capturing complementary computational demands:

(1) categorical accuracy—whether the displayed target emotion received the highest rating among six emotion categories (i.e., *did participants correctly identify the dominant emotion?*);
(2) graded tolerance accuracy—the proportion of ratings falling within ±2 intensity levels of the correct value (i.e., *how often were participants approximately correct, even if not exact?*); and
(3) mean absolute error—the absolute distance between the rated and true intensity (i.e., *how precisely could participants match the true emotional strength?*).

2. Supplementary Results

2.1 Study 2 (Congenital Facial Palsy)

2.1.1 Behavioral Results

A GLMM predicting recognition accuracy from expression type (ADFES vs. JeFEE), group (Moebius vs. control), and their interaction revealed a robust main effect of expression type: JeFEE were significantly harder to recognize than ADFES, $\beta = -1.33$, $SE = 0.12$, $z = -11.17$, $p < 0.001$, $d = 0.74$. There was no main effect of group (Moebius vs. control), $\beta = 0.01$, $SE = 0.20$, $z = 0.06$, $p = 0.95$, and no interaction between expression type and group, $\beta = -0.04$, $SE = 0.17$, $z = -0.24$, $p = 0.81$. See Supplementary Fig. 2, Panel A. Thus, recognition accuracy was consistently higher for prototypical expressions, with no evidence that overall accuracy or the prototypicality effect differed between groups. Random intercepts for participants captured individual differences in baseline accuracy ($SD = 0.43$).

Perceived intensity also showed a strong main effect of expression type ($F_{1,28} = 58.93$, $p < 0.001$, $\eta_p^2 = 0.68$), with higher ratings for ADFES than JeFEE expressions. No main effect of group was observed ($F_{1,28} = 0.30$, $p = 0.59$, $\eta_p^2 = 0.01$). However, a significant interaction ($F_{1,28} = 4.30$, $p = 0.047$, $\eta_p^2 = 0.13$) indicated a larger ADFES–JeFEE intensity difference in the Moebius group (Supplementary Fig. 2, Panel B).

Post-hoc Welch's t-tests confirmed that ADFES accuracy ($t_{26.36} = -0.03$, $p = 0.97$; $d = -0.01$) and intensity ($t_{27.97} = -0.02$, $p = 0.98$; $d = -0.008$) did not differ significantly between groups, nor did JeFEE accuracy ($t_{27.41} = 0.16$, $p = 0.88$; $d = 0.06$) or JeFEE intensity ($t_{27.22} = 0.98$, $p = 0.33$; $d = 0.36$).

Bayes factors provided strong evidence for expression type effects ($BF_{10} > 100$), moderate support for the null hypothesis regarding group differences in accuracy and ADFES

intensity (BF_{01} = 2.10), and strong evidence for the interaction in intensity ratings (BF_{10} > 100). Cohen's d indicated negligible group differences for accuracy and ADFES intensity (d ≈ 0.01–0.06), and a small-to-moderate effect for JeFEE intensity (d = 0.36).

In the Moebius group, expression type (prototypical vs. non-prototypical) strongly modulated both recognition accuracy and perceived intensity, with no significant differences from matched controls except for a modest interaction effect in intensity ratings.

Compared to the reference sample (n = 117; Study 1), both the Moebius and control groups showed reduced accuracy. ADFES accuracy was marginally lower in controls (t_{14} = -1.54, p = 0.15, d = -0.40, BF_{10} = 0.69) and Moebius patients (t_{14} = -1.93, p = 0.08, d = -0.50, BF_{10} = 1.13). JeFEE accuracy was significantly reduced in both groups (controls: t_{14} = -2.26, p = 0.04, d = -0.58, BF_{10} = 1.81; Moebius: t_{14} = -2.15, p = 0.05, d = -0.56, BF_{10} = 1.56). The magnitude of these decrements was comparable between groups for both ADFES (z = 0.26, p = 0.79) and JeFEE (z = -0.07, p = 0.95).

Intensity ratings showed significant reductions in both groups for ADFES (controls: t_{14} = -5.35, p < 0.001, d = -1.38; Moebius: t_{14} = -5.49, p < 0.001, d = -1.42) and JeFEE expressions (controls: t_{14} = -3.61, p = 0.002, d = -0.93; Moebius: t_{14} = -4.33, p < 0.001, d = -1.12). The magnitude of these intensity decrements was comparable between groups for both ADFES (z = 0.08, p = 0.94) and JeFEE (z = 0.41, p = 0.68).

Effect sizes revealed robust intensity decrements (d = -0.93 to -1.42) and moderate accuracy reductions (d = -0.40 to -0.58). Between-group comparisons showed no differences in either accuracy or intensity decrements (all |z| ≤ 0.41, all p ≥ 0.68). This pattern suggests that both the Moebius and control groups perceive expressions as less vivid than norms, while maintaining relatively preserved recognition abilities.

No group differences were observed on AQ, TAS-20, or L-POST (all $p > 0.2$) between patients and their matched controls.

2.1.2 Mediation Analyses

Exploratory mediation analyses tested whether alexithymic (TAS) or autistic (AQ) traits could account for the observed association between facial motor function and emotion recognition, thereby providing a potential alternative explanation for the severity–performance coupling. We acknowledge these analyses are underpowered (29). Despite these limitations, we report them for transparency and to inform future research planning. With FDR control ($q = 0.05$), the total effect of Sunnybrook score remained significant for both ADFES ($\beta = 0.0034$, 95% CI [0.0001, 0.0070], $q = 0.044$) and JeFEE ($\beta = 0.0061$, 95% CI [0.0010, 0.0112], $q = 0.036$). SFGS scores did not predict TAS ($\beta = 0.028$, 95% CI [–0.30, 0.35], $q = 0.86$) or AQ ($\beta = 0.09$, 95% CI [–0.13, 0.31], $q = 0.78$). Correspondingly, all bias-corrected indirect (ACME) effects were minute and non-significant ($-0.034 < \beta < 0.036$; $q \approx 0.99$). Given the minimal sample, the power to detect an indirect effect smaller than $|\beta| \approx 0.03$ is < 40%; around 120 participants would be required for 80% power. Within the present data, alexithymic and autistic traits do not account for the motor–perceptual association.

Palsy laterality, age, years of education, and gender showed no statistically reliable associations with emotion-recognition accuracy after FDR correction (all $q \geq 0.89$). Even the largest observed association—education versus JeFEE accuracy ($r = 0.46$, $p = 0.089$, $q = 0.89$)—failed to survive FDR correction and carried only anecdotal evidence in favor of an effect ($BF_{10} = 1.50$). Thus, within this sample, no clinical or demographic factor meaningfully explains variance in emotion-recognition performance.

2.2 Study 3 (Acquired Facial Palsy)

A GLMM predicting recognition accuracy from expression type (ADFES vs. JeFEE), group (acquired facial palsy vs. control), and their interaction revealed a strong main effect of expression type: recognition was significantly lower for JeFEE than for ADFES, $\beta = -1.19$, $SE = 0.10$, $z = -11.63$, $p < 0.001$, $d = 0.65$. There was no main effect of group, $\beta = 0.26$, $SE = 0.19$, $z = 1.35$, $p = 0.178$, and no expression × group interaction, $\beta = 0.02$, $SE = 0.14$, $z = 0.14$, $p = 0.887$, indicating that both groups showed similar performance and similar prototypicality effects (Supplementary Fig. 4, Panel A). Random intercepts for participants ($SD = 0.49$) accounted for individual variability in baseline recognition accuracy.

Follow-up Welch's t-tests revealed no significant group differences for ADFES accuracy ($t_{27.44} = -1.25$, $p = 0.22$, $d = 0.41$, $BF_{10} = 0.58$), ADFES intensity ($t_{29.29} = 0.52$, $p = 0.603$, $d = 0.17$, $BF_{10} = 0.35$), or JeFEE accuracy ($t_{35.63} = 1.44$, $p = 0.157$, $d = 0.47$, $BF_{10} = 0.71$). In contrast, the palsy group rated JeFEE expression intensity significantly lower than controls ($t_{33.27} = 2.36$, $p = 0.024$, $d = 0.77$, $BF_{10} = 2.62$), providing a weak-to-moderate Bayesian support for this difference (Supplementary Fig. 4A-B).

Statistical evidence robustly supports a main effect of expression type ($d = 0.65$ for accuracy, $\eta_p^2 = 0.59$ for intensity) and a significant expression × group interaction for intensity ($\eta_p^2 = 0.19$). Effect sizes were moderate for accuracy ($d = 0.41 – 0.47$) and moderate-to-large for JeFEE intensity ($d = 0.77$; $BF_{10} = 2.62$), indicating that facial palsy primarily reduces the perceived intensity of non-prototypical expressions, while having minimal impact on recognition accuracy or perception of more prototypical expressions.

Comparison with the reference sample ($n = 117$; Study 1) using one-sample t-tests against the reference-cohort mean revealed significant accuracy decrements in both groups,

particularly for prototypical expressions. ADFES accuracy was lower than the reference in controls ($t_{18} = -3.71$, $p = 0.002$, $d = -0.85$, $BF_{10} = 24.56$) and palsy patients ($t_{18} = -4.30$, $p = 0.001$, $d = -0.99$, $BF_{10} = 77.10$). Similarly, JeFEE accuracy showed decrements in both groups (controls: $t_{18} = -4.67$, $p < 0.001$, $d = -1.07$, $BF_{10} = 159.70$; palsy: $t_{18} = -3.02$, $p = 0.007$, $d = -0.69$, $BF_{10} = 6.74$). The magnitude of these decrements did not differ between groups (ADFES: $z = 0.41$, $p = 0.67$; JeFEE: $z = -1.16$, $p = 0.24$), indicating comparable impairments relative to the reference cohort.

For intensity ratings, controls showed no reliable reductions for ADFES ($t_{18} = -0.31$, $p = 0.759$, $d = -0.07$, $BF_{01} = 4.03$) or JeFEE ($t_{18} = 0.61$, $p = 0.55$, $d = 0.14$, $BF_{01} = 3.55$). The palsy group showed a trend for reduced ADFES intensity ($t_{18} = -1.55$, $p = 0.138$, $d = -0.36$, $BF_{01} = 1.51$) and significantly lower JeFEE intensity ratings ($t_{18} = -3.14$, $p = 0.005$, $d = -0.72$, $BF_{10} = 8.31$).

Bayes factors provided moderate-to-strong evidence for accuracy decrements relative to the reference cohort ($BF_{10} = 6.74$–159.70) and moderate evidence for reduced JeFEE intensity in the acquired-palsy group ($BF_{10} = 8.31$). The magnitude of the accuracy decrements did not differ significantly between patients and matched controls (ADFES: $z = 0.41$, $p = 0.67$; JeFEE: $z = -1.16$, $p = 0.24$). The palsy group showed significantly greater impairment in intensity perception for non-prototypical expressions, suggesting that facial paralysis specifically affects the experiential dimension of emotion recognition while preserving categorical judgments.

2.2.2 Mediation Analyses

The following mediation analyses face similar power limitations as noted earlier (29). We include them here for completeness and to guide sample size planning in subsequent

research. These exploratory mediation analyses were conducted to test whether depressive symptoms (MADRS) provide an alternative explanation for the association between facial motor function and emotion recognition performance. For JeFEE accuracy, the total effect of the SFGS score remained significant after FDR control ($\beta = 0.0035$, 95 % BCa CI [0.0014, 0.0053], $q = 0.014$). The direct effect was likewise significant ($\beta = 0.0034$, 95 % CI [0.0011, 0.0057], $q = 0.019$), whereas the bias-corrected indirect effect via MADRS was negligible and non-significant (ACME = 0.00006, 95 % CI [–0.0010, 0.0010], $q \approx 0.97$). For ADFES accuracy, the total effect of the SFGS score was statistically significant after FDR correction ($\beta = 0.00176$, 95% CI [0.00012, 0.00365], $q = 0.0384$). However, the indirect effect through MADRS was negligible and non-significant (ACME = 0.00011, 95% CI [–0.00052, 0.00132], $q = 0.99$). Within the constraints of our sample size, depressive symptoms do not appear to account for the association between facial motor function and emotion-recognition accuracy.

Side of palsy, years since onset, etiology, and gender showed no detectable relationships with emotion-recognition accuracy (all two-sided $p \geq 0.09$, $q = 0.82$, $|d| \leq 0.72$; $BF_{01} \geq 2.27$). While these variables do not appear to drive performance variation in our sample, larger studies would be needed to rule out small to moderate effects.

Linear models confirmed that SFGS scores did not predict perceived-intensity ratings (JeFEE: $R^2 = 0.01$, $\beta = 0.14 \pm 0.36$, $p = 0.65$, $BF_{01} = 2.3$; ADFES: $R^2 = 0.01$, $\beta = –0.11 \pm 0.25$, $p = 0.66$, $BF_{01} = 2.4$). Patients nevertheless judged JeFEE expressions as less intense than controls ($M = 137.3 \pm 27.8$ vs 162.6 ± 37.4; $d = 0.77$, 95 % BCa CI [0.22, 0.81], $p = 0.023$, $q = 0.047$, $BF_{10} = 2.62$) and showed a compressed intensity range, with only 2/19 (10.5 %) reaching the control mean. No difference emerged for ADFES intensity ($p = 0.60$). Mediation analysis with 10,000 BCa bootstraps confirmed that perceived intensity did not transmit the effect of facial motor function on JeFEE accuracy (ACME = –0.0000012, 95 % BCa CI [–0.000615,

0.00166], $p = 0.69$, $BF_{01} = 0.99$). Thus, the association between residual motor function and emotion-recognition accuracy does not appear to be accounted for by depressive symptoms.

2.3 Summary of Patient–Control Behavioral Comparisons

Both Studies 2 and 3 employed three-cohort comparisons: patients with facial paralysis (congenital, $n = 15$; acquired, $n = 19$), demographically matched controls ($n = 15$ and $n = 19$, respectively), and a reference large sample ($n = 117$; from Study 1). This design enabled us to isolate paralysis-specific effects while accounting for demographic factors and normal performance variability. Across all three cohorts, recognition accuracy followed the same robust pattern: prototypical ADFES expressions were identified far more readily than non-prototypical JeFEE expressions across studies ($d = 0.65$–0.76). Neither Moebius nor acquired-palsy patients differed from their matched controls, and no group × prototypicality interaction approached significance (Moebius $p = 0.81$; acquired $p = 0.887$). Both laboratory samples (patients and controls) scored slightly below the larger reference cohort's benchmark to an equal extent, suggesting a contextual—rather than clinical—effect.

Perceived intensity diverged by etiology. In Moebius syndrome, a small but significant interaction was reflected in a marginally wider ADFES–JeFEE gap, yet absolute intensity ratings for each set matched those of the controls. In acquired palsy, by contrast, participants downrated the vividness of non-prototypical JeFEE clips ($d \approx 0.8$) while judging ADFES normally, yielding a clearer interaction that pinpoints post-developmental motor loss as the driver of this phenomenological shift.

2.4 Intensity-Rating Task (ref. 27): Between-Group Results

Categorical accuracy index. A binomial generalized linear model revealed a significant main effect of the group ($\chi^2(2) = 28.41$, $p < 0.001$). Post-hoc comparisons (Bonferroni-corrected) showed that participants in the Mask condition performed significantly worse than Controls (OR = 1.93, p = 0.003), and Moebius participants performed significantly worse than Controls (OR = 2.81, p < 0.001), whereas the Moebius–Mask contrast was not significant (p = 0.13). Thus, acute facial movement restriction in neurotypical adults reduced categorical accuracy to levels approaching those of congenital facial paralysis.

Graded tolerance accuracy. A binomial generalized linear model revealed a significant main effect of the group ($\chi^2(2) = 12.59$, $p = 0.0018$). Post-hoc comparisons showed that Mask participants performed slightly worse than Controls, though this difference did not reach significance ($OR = 1.29$, $p = 0.28$). Moebius participants performed significantly worse than Controls ($OR = 1.68$, $p = 0.002$), whereas the Mask–Moebius contrast was not significant ($p = 0.44$). Mask participants showed a small, non-significant reduction in graded tolerance accuracy relative to Controls, whereas Moebius participants showed a significant decrement, consistent with a greater sensorimotor contribution under high perceptual demand.

Mean absolute error. A Gaussian linear model showed no significant main effect of the group ($F(2, 30) = 2.00$, $p = 0.15$). Although Moebius participants tended to show higher mean distance scores than Controls ($\beta = 0.48$, $p = 0.06$), this trend did not reach significance. No difference emerged between Mask and Control groups ($p = 0.61$). These results suggest that while congenital facial paralysis may slightly reduce precision in continuous intensity estimation, group effects were overall modest compared to categorical and graded accuracy metrics.

2.5 Intensity-Rating Task (ref. 27): Severity–Performance Correlations in the Moebius Group

Within the Moebius sample, higher facial motor function (SFGS score) was associated with better performance across all behavioural metrics. Spearman correlations confirmed significant coupling for graded tolerance accuracy ($\rho = 0.68$, $p = 0.031$) and mean distance ($\rho = –0.71$, $p = 0.025$), with marginal associations for categorical recognition ($\rho = 0.61$, $p = 0.054$) and accuracy within ±1 intensity level ($\rho = 0.51$, $p = 0.096$). Effect sizes were larger for graded than for categorical metrics, suggesting that continuous measures may be more sensitive to individual differences in performance under high perceptual demand.

2.6 Robustness of the aggregate recognition score across emotion categories

Rationale. The principal analyses relate SFGS scores to recognition accuracy aggregated across the six basic emotions. To verify that this aggregate faithfully reflects broad recognition performance rather than heterogeneous, emotion-specific profiles, we performed two complementary checks. We treat both as descriptive robustness analyses, not as emotion-specific tests: with eight trials per emotion per stimulus set, single-emotion estimates are imprecise, and we draw no inference from them.

Leave-one-emotion-out (LOEO). We excluded each of the six emotions in turn, recomputed aggregate accuracy from the remaining five (≈40 trials per set), and re-estimated its Pearson correlation with SFGS. This analysis tests whether the observed severity–performance relationship depends disproportionately on any single emotion category. If so, excluding that emotion should substantially attenuate the correlation. This prediction was not supported. Across every cohort and stimulus set, the severity–performance coupling remained positive throughout all LOEO iterations (Table S3):

- Moebius, ADFES: full $_r$ = 0.62, LOEO $_{range}$ 0.51–0.63
- Moebius, JeFEE: full $_r$ = 0.64, LOEO $_{range}$ 0.46–0.71
- Acquired, JeFEE: full $_r$ = 0.60, LOEO $_{range}$ 0.51–0.63
- Acquired, ADFES: full $_r$ = 0.41 (the weakest, non-significant association in the main analyses), LOEO $_{range}$ 0.27–0.45

The largest single-emotion attenuations were observed for anger in the Moebius JeFEE set (Δr = 0.19) and disgust in the acquired ADFES set (Δr = 0.14). Even in these cases the coupling remained positive, and no single emotion was necessary to produce it.

Inter-emotion covariation (descriptive). For completeness, we examined pairwise correlations among the six emotion-specific accuracy scores within each cohort and stimulus set, together with the variance explained by the first principal component. Pairwise correlations were generally positive but modest (mean r = 0.04–0.19), and the first principal component accounted for 29–35% of the variance. Given the limited number of trials per emotion (eight per stimulus set) and the small clinical samples, these analyses are presented for descriptive purposes only and are not interpreted further. Accordingly, we base no inference on these estimates; the leave-one-emotion-out analysis reported above provides the primary evidence that the aggregate recognition score is not disproportionately driven by any single emotion category.

Supplementary Fig. 7 provides a descriptive visualization of selected severity–performance relationships across etiologies.

3. Supplementary Tables

Demographic and clinical characteristics of participants with congenital (Supplementary Table 1) and acquired (Supplementary Table 2) facial palsy. Study 2 includes patients with congenital facial palsy (Moebius), while Study 3 includes patients with acquired facial palsy from various etiologies. SFGS scores range from 0 to 100, with higher scores indicating better facial function. Years since onset represents the time from initial symptom presentation to study enrollment for acquired cases.

Supplementary Table 1. **Demographic & Clinical Characteristics — Study 2**

**Congenital Facial Palsy (n=15)**					
**ID**	**Sex**	**Age (yrs)**	**Education (yrs)**	**SFGS**	**Affected Laterality**
**CFP1**	F	22	17	12	Bilateral
**CFP2**	M	56	8	17	Bilateral
**CFP3**	F	66	17	21	Bilateral
**CFP4**	M	38	17	27	Bilateral
**CFP5**	M	23	13	30	Unilateral (Left)
**CFP6**	F	32	17	35	Bilateral
**CFP7**	F	40	17	51	Unilateral (Right)
**CFP8**	F	26	17	54	Unilateral (Right)
**CFP9**	F	18	13	17	Bilateral
**CFP10**	M	51	17	44	Bilateral
**CFP11**	F	32	17	33	Unilateral (Right)
**CFP12**	M	57	15	40	Unilateral (Left)
**CFP13**	M	19	15	48	Bilateral
**CFP14**	F	27	15	76	Unilateral (Right)
**CFP15**	M	22	15	49	Bilateral

Supplementary Table 2. **Demographic & Clinical Characteristics — Study 3**

**Acquired Facial Palsy (n=19)**							
**ID**	**Sex**	**Age (yrs)**	**Education (yrs)**	**SFGS**	**Affected Laterality**	**Etiology**	**Onset (yrs)**
**AFP1**	F	43	14	39	Left	Iatrogenic: I-VS	5
**AFP2**	F	50	13	50	Left	Iatrogenic: I-VS	7
**AFP3**	F	52	13	45	Right	Idiopathic: BP	13
**AFP4**	F	37	19	44	Right	Vascular: PCA	5
**AFP5**	F	59	19	56	Right	Iatrogenic: I-VS	8
**AFP6**	F	62	17	41	Left	Iatrogenic: I-M	3
**AFP7**	M	19	13	36	Right	Iatrogenic: I-MC	5
**AFP8**	M	53	15	19	Left	Idiopathic: BP	15
**AFP9**	F	39	14	89	Right	Viral: RH	7
**AFP10**	F	61	17	87	Left	Viral: RH	8
**AFP11**	F	41	16	65	Left	Idiopathic: BP	4
**AFP12**	F	56	13	56	Right	Vascular: ACH	5
**AFP13**	F	29	18	44	Right	Iatrogenic: I-N	11
**AFP14**	M	45	13	32	Left	Vascular: MAV	3
**AFP15**	F	52	13	90	Left	Iatrogenic: I-VS	5

Acquired Facial Palsy (n=19)							
**ID**	**Sex**	**Age (yrs)**	**Education (yrs)**	**SFGS**	**Affected Laterality**	**Etiology**	**Onset (yrs)**
**AFP16**	F	19	13	74	Left	Inflammatory: MRN	16
**AFP17**	F	67	11	78	Right	Idiopathic: BP	5
**AFP18**	M	38	13	80	Left	Iatrogenic: I-VS	2
**AFP19**	F	48	13	43	Right	Viral: Z	11

Etiology abbreviations: Idiopathic: BP = Bell's Palsy; **Iatrogenic:** I-VS = Iatrogenic (Vestibular Schwannoma), I-M = Iatrogenic (Meningioma), I-MC = Iatrogenic (Cerebellar Medulloblastoma), I-N = Iatrogenic (Neurinoma); **Viral:** RH = Ramsay Hunt, Z = Zoster; **Vascular:** PCA = Pontine Cavernous Angioma, ACH = Angioma Cavernoso + Emorragia Cerebrale Bulbo Pontina, MAV = MAV Rocca Petrosa; **Inflammatory:** MRN = HBV-related Mieloradicolonevrite.

Supplementary Table 3. Leave-one-emotion-out analysis of the aggregate recognition score — Study 2 and 3

Study 2					
**Cohort**	**Stimulus set**	**Full $_r$**	**LOEO $_{range}$**	**Emotion excluded**	**Max Δ*r***
Congenital facial palsy	ADFES	0.62	0.51-0.63	Happiness	0.11
	JeFEE	0.64	0.46-0.71	Anger	0.19
Study 3					
Acquired facial palsy	ADFES	0.41	0.27-0.45	Disgust	0.14
	JeFEE	0.60	0.51-0.63	Surprise	0.10

Aggregate recognition score validity. Full $_r$ = Pearson correlation between SFGS score and aggregate recognition accuracy computed across all six emotions. LOEO $_{range}$ = minimum and maximum Pearson correlations obtained across the six leave-one-emotion-out iterations. Excluded emotion = emotion whose exclusion produced the largest attenuation of the correlation. Max Δr = Full r − minimum LOEO correlation. LOEO analyses were not performed for Study 1 because SFGS scores were unavailable. Differences were calculated from unrounded correlations and may differ slightly from differences between the displayed rounded values.

4. Supplementary Figures

Supplementary Fig. 1. **Geneva Emotion Wheel (Italian Translation)**

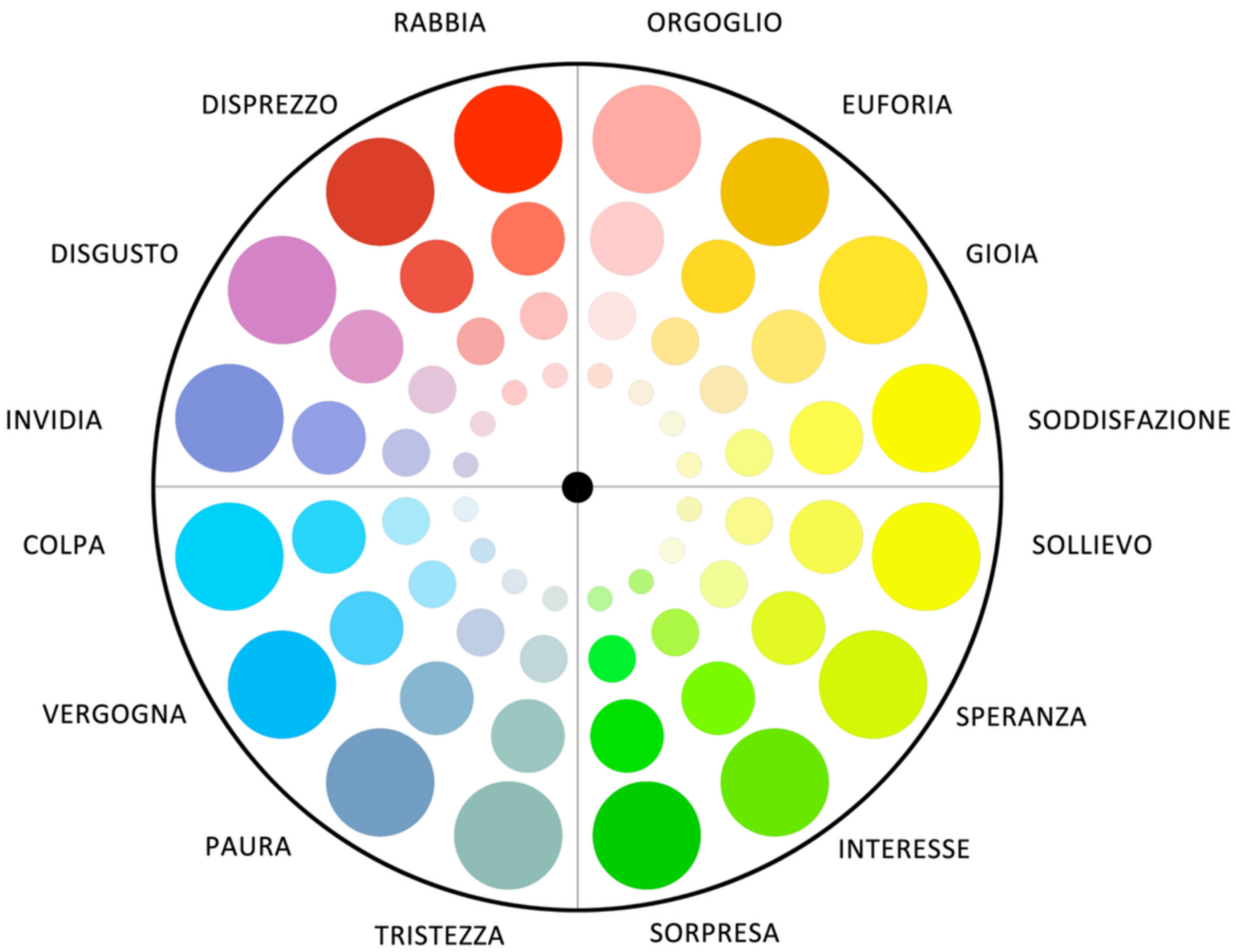

Supplementary Fig. 1. Prototype of GEW (Geneva Emotion Wheel, Version 1.0) featuring 16 emotional terms (based on the GEW 1.0 described in ref. 10).

Supplementary Fig. 2. **Accuracy and Perceived Intensity, group comparisons (Study 2)**

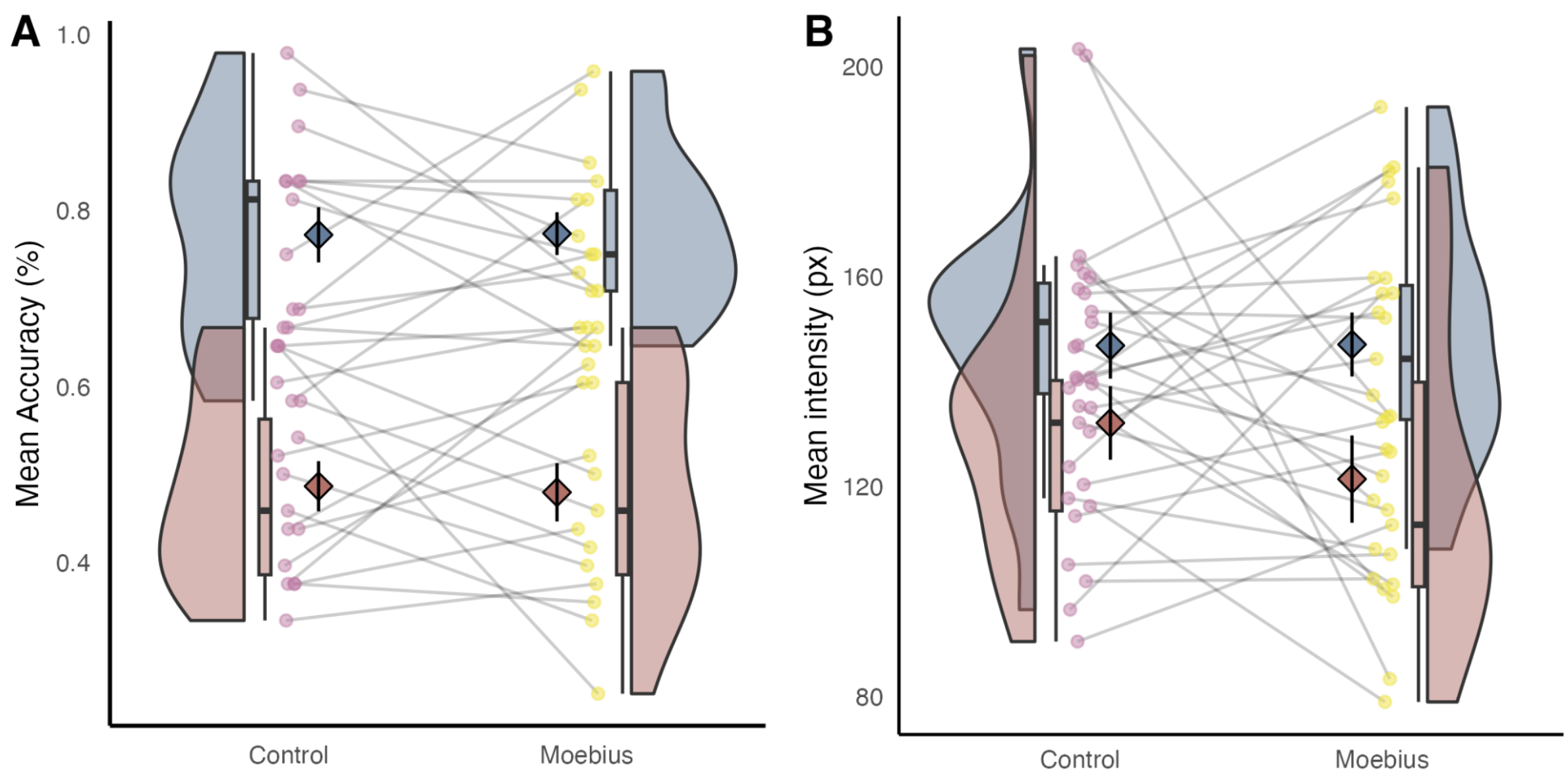

Supplementary Fig. 2. Recognition accuracy (A) and perceived intensity (B) for prototypical (ADFES, blue) and non-prototypical (JeFEE, red) facial expressions in Moebius syndrome (n = 15) and matched controls (n = 15). Violin plots show individual mean scores, with boxplots indicating interquartile range and median, and black diamonds denoting group means ± standard error.

***Supplementary Fig. 3.* SFGS–accuracy relationship by facial lateralisation**

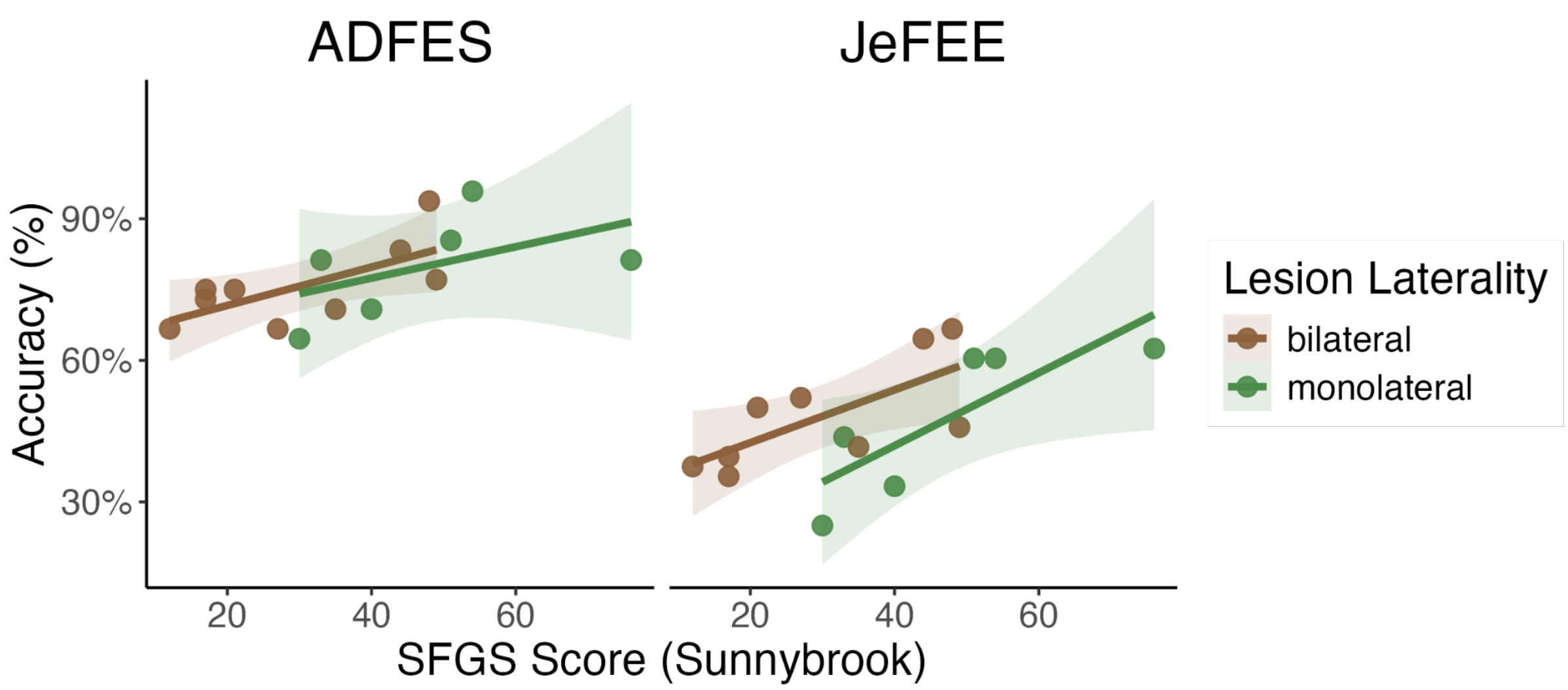

Supplementary Fig. 3. Relationship between SFGS scores and emotion-recognition accuracy (%) for ADFES (left) and JeFEE (right) expressions in participants with congenital facial palsy, stratified by facial laterality. Brown points and fitted lines denote bilateral involvement, and green points and fitted lines denote monolateral involvement; shaded areas indicate 95% confidence intervals. Laterality did not interact with SFGS ($p = 0.81$).

Supplementary Fig. 4. **Accuracy and Perceived Intensity, group comparisons (Study 3)**

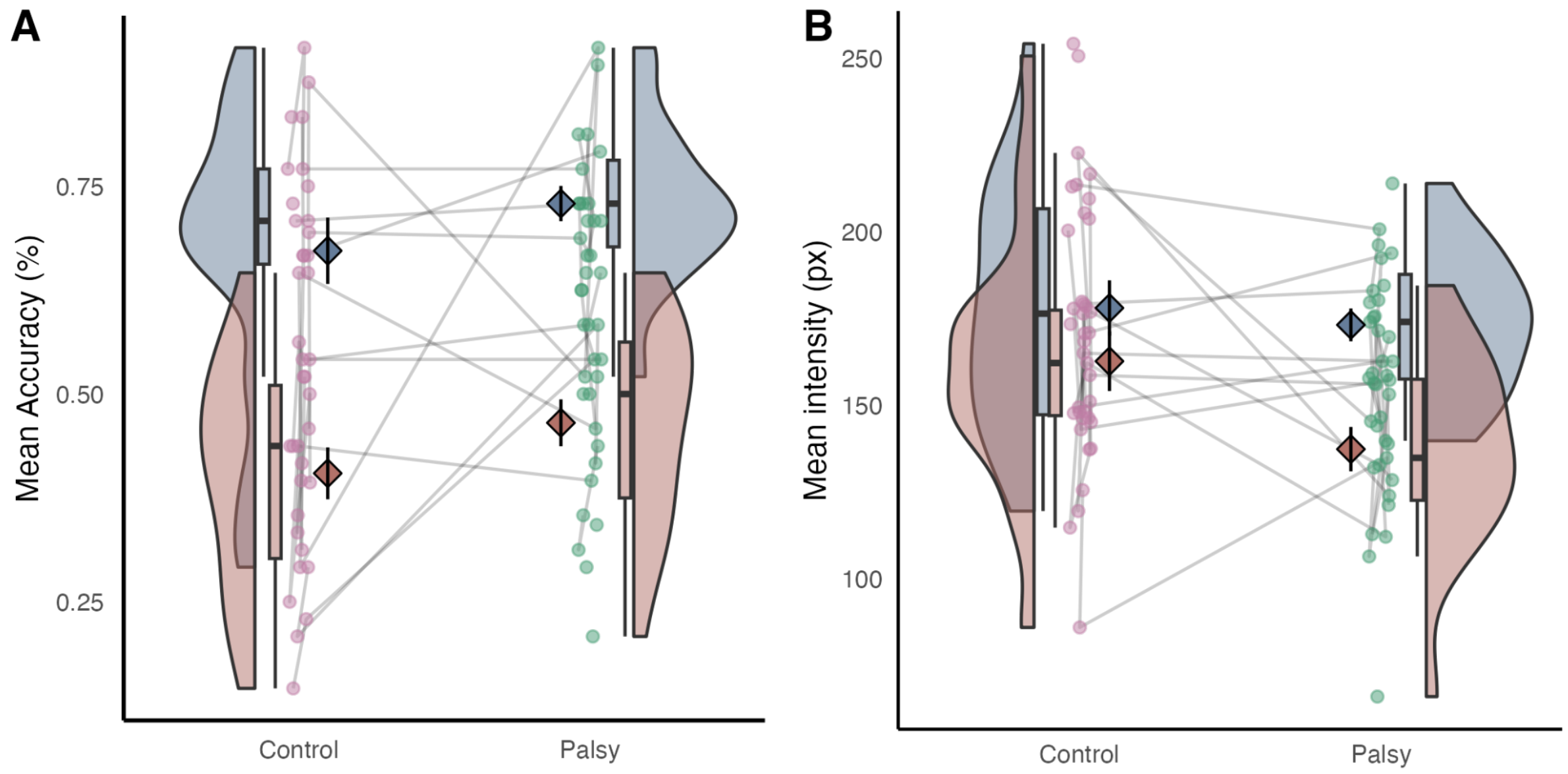

Supplementary Fig. 4. Recognition accuracy (A) and perceived intensity (B) for prototypical (ADFES, blue) and non-prototypical (JeFEE, red) facial expressions in acquired facial palsy (n = 19) and matched controls (n = 19). Violin plots show individual mean scores, with boxplots indicating interquartile range and median, and black diamonds denoting group means ± standard error.

Supplementary Fig. 5. Group comparison – Morphed Intensity-Rating (27)

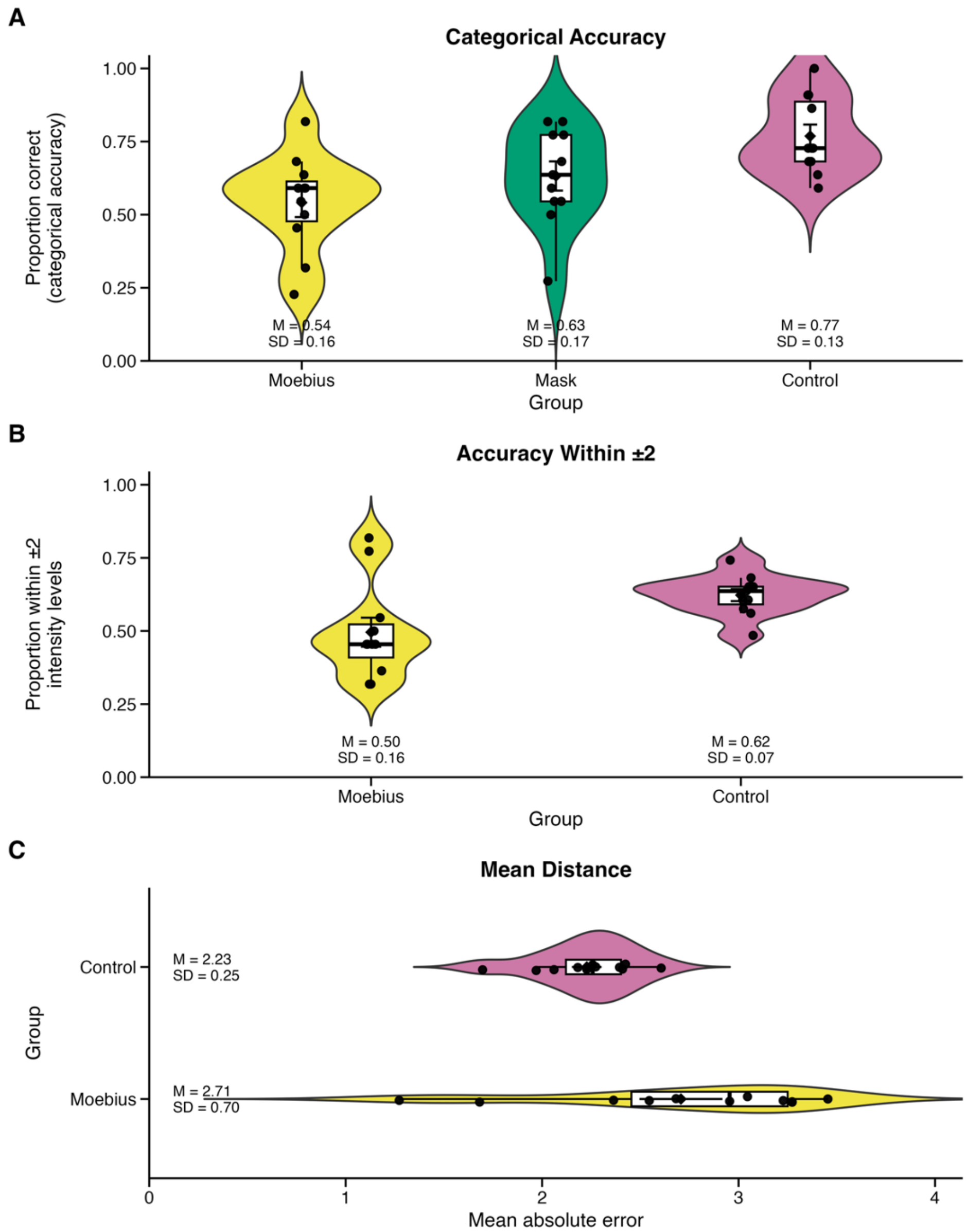

Supplementary Fig. 5. Recognition accuracy in the Morphed Intensity-Rating task. (A) Categorical accuracy across Control, Mask, and Moebius groups. (B) Proportion of ratings within ±2 intensity levels from the true target (Control vs. Moebius). (C) Mean absolute distance between rated and true intensity (Control vs. Moebius). Violin plots display individual mean scores, overlaid with boxplots indicating interquartile range and median; black diamonds denote group means ± standard error.

Supplementary Fig. 6. Correlations between facial motor function (SFGS) and emotion recognition performance in Moebius syndrome

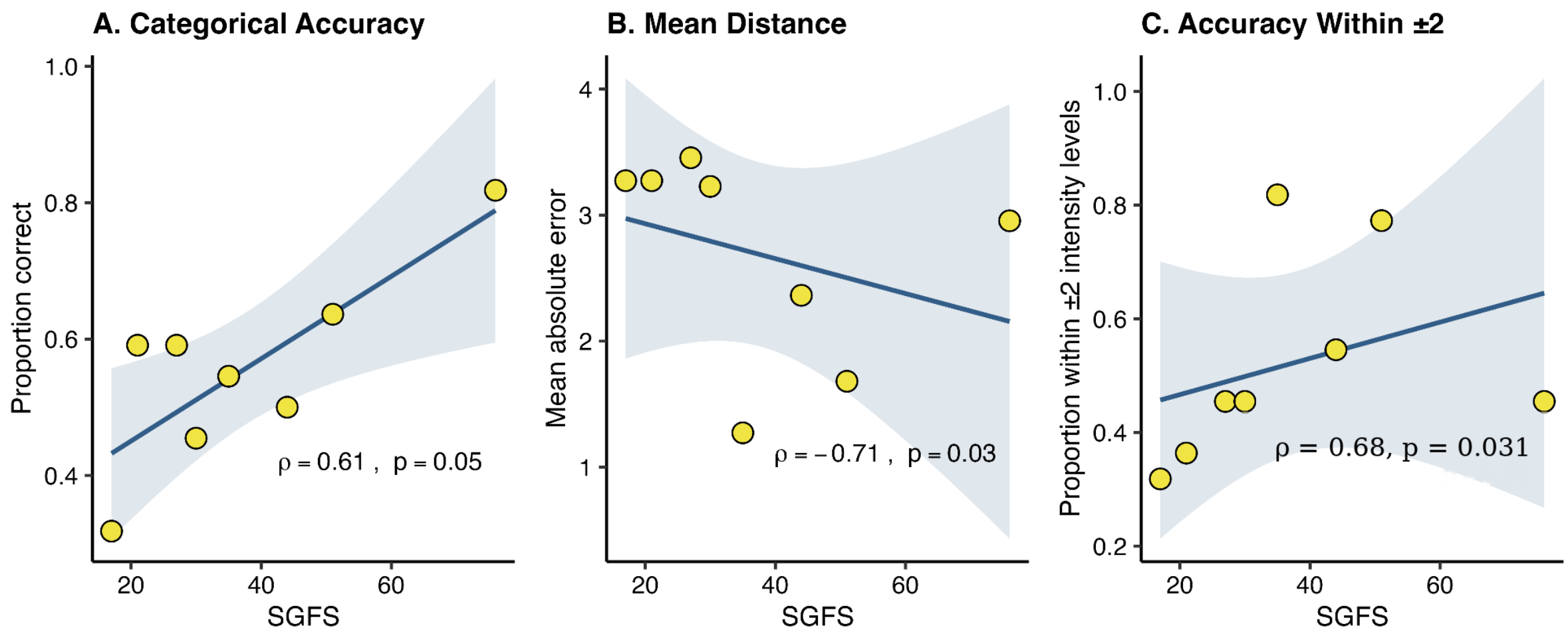

Supplementary Fig. 6. Correlations between SFGS scores and three performance indices in Moebius participants (n = 8) in the Morphed Intensity-Rating task (27). (A) Categorical accuracy (proportion of correctly identified target emotions). (B) Mean absolute error between rated and true emotion intensity. (C) Proportion of ratings within ±2 intensity levels from the true target. Shaded areas indicate 95% confidence intervals for the regression line; ρ and *p* values denote one-tailed Spearman correlations.

Supplementary Fig. 7. Exploratory visualization of severity–performance relationships across etiologies

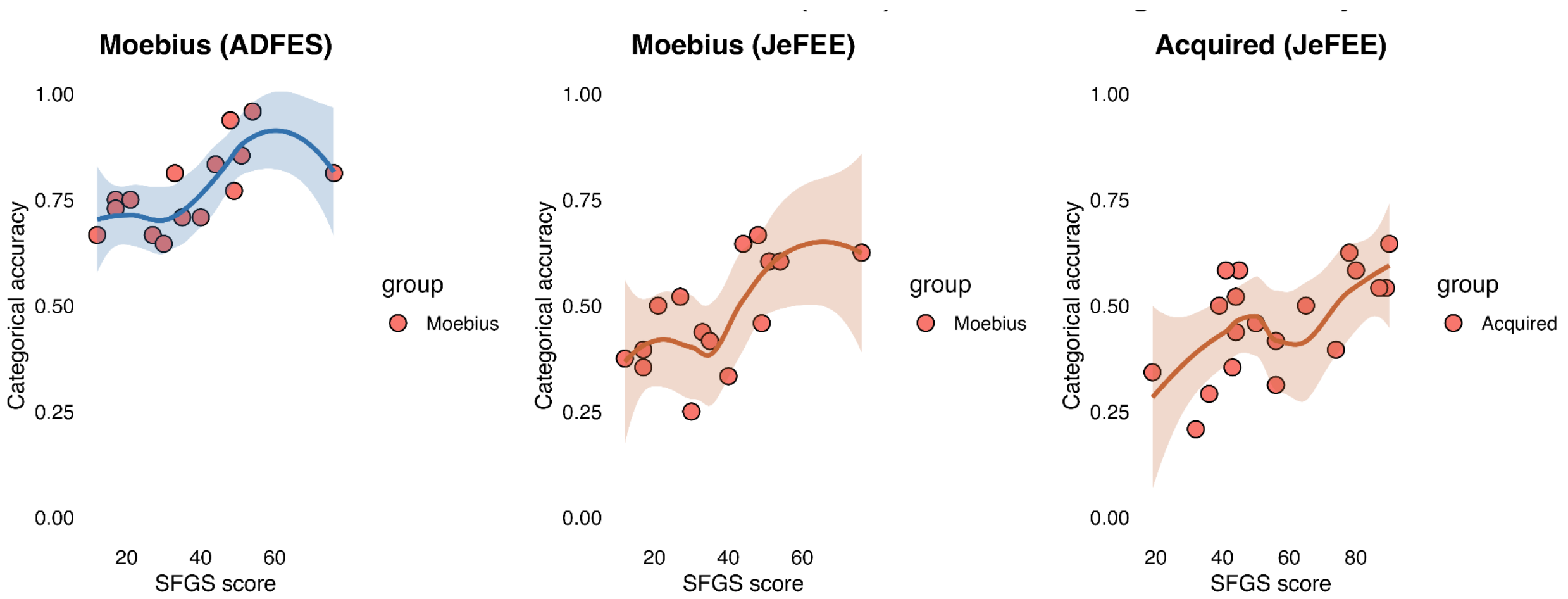

Supplementary Fig. 7. Each dot represents an individual participant. LOESS-smoothed curves (span = 0.75) with pointwise 95% confidence bands are shown for the two Moebius stimulus sets and for acquired-palsy JeFEE performance. Given the small clinical samples, these curves are presented for descriptive purposes only and are not used to infer differences in functional form. The acquired-palsy ADFES pairing is omitted here because its severity–performance association was the weakest and non-significant among the four cohort-by-stimulus-set combinations (r = 0.41, Section 2.6).

5. Supplementary References

1. Pavese, C. et al. Validation of the Italian version of the Sunnybrook Facial Grading System. Neurol. Sci. 34, 457–463 (2013).
2. Ross, B. G., Fradet, G. & Nedzelski, J. M. Development of a sensitive clinical facial grading system. Otolaryngol. Head Neck Surg. 114, 380–386 (1996).
3. Bagby, R. M., Parker, J. D. & Taylor, G. J. The twenty-item Toronto Alexithymia Scale—I. item selection and cross-validation of the factor structure. J. Psychosom. Res. 38, 23–32 (1994).
4. Baron-Cohen, S. et al. The Autism-Spectrum Quotient (AQ): evidence from Asperger syndrome/high-functioning autism, males and females, scientists and mathematicians. J. Autism Dev. Disord. 31, 5–17 (2001).
5. Montgomery, S. A. & Åsberg, M. A new depression scale designed to be sensitive to change. Br. J. Psychiatry 134, 382–389 (1979).
6. Stantic, M. et al. The Oxford Face Matching Test: a non-biased test of the full range of individual differences in face perception. Behav. Res. Methods 54, 158–173 (2022).
7. Torfs, K., Vancleef, K., Lafosse, C., Wagemans, J. & De-Wit, L. The Leuven Perceptual Organization Screening Test (L-POST), an online test to assess mid-level visual perception. Behav. Res. Methods 46, 472–487 (2014).
8. van der Schalk, J., Hawk, S. T., Fischer, A. H. & Doosje, B. J. Moving faces, looking places: validation of the Amsterdam Dynamic Facial Expression Set (ADFES). Emotion 11, 907–920 (2011).
9. Yitzhak, N. et al. Gently does it: humans outperform a software classifier in recognizing subtle, non-stereotypical facial expressions. Emotion 17, 1187–1198 (2017).

10. Scherer, K. R. What are emotions? And how can they be measured? Soc. Sci. Inf. 44, 693–727 (2005).

11. Scherer, K. R., Shuman, V., Fontaine, J. R. J. & Soriano, C. The GRID meets the Wheel: assessing emotional feeling via self-report. In Components of Emotional Meaning: A Sourcebook (eds Fontaine, J. R. J., Scherer, K. R. & Soriano, C.) 281–298 (Oxford Univ. Press, 2013).

12. Russell, J. A. A circumplex model of affect. J. Pers. Soc. Psychol. 39, 1161–1178 (1980).

13. Anwyl-Irvine, A. L., Massonnié, J., Flitton, A., Kirkham, N. & Evershed, J. K. Gorilla in our midst: an online behavioral experiment builder. Behav. Res. Methods 52, 388–407 (2020).

14. de Cheveigné, A. ZapLine: a simple and effective method to remove power line artifacts. NeuroImage 207, 116356 (2020).

15. Pion-Tonachini, L., Kreutz-Delgado, K. & Makeig, S. ICLabel: an automated electroencephalographic independent component classifier, dataset, and website. NeuroImage 198, 181–197 (2019).

16. Foxe, J. J. & Snyder, A. C. The role of alpha-band brain oscillations as a sensory suppression mechanism during selective attention. Front. Psychol. 2, 154 (2011).

17. Klimesch, W. Alpha-band oscillations, attention, and controlled access to stored information. Trends Cogn. Sci. 16, 606–617 (2012).

18. Genzer, S. et al. Mu rhythm suppression over sensorimotor regions is associated with greater empathic accuracy. Soc. Cogn. Affect. Neurosci. 17, 788–801 (2022).

19. Birch-Hurst, K., Rafiei, R., Lewis, M. B., Rychlowska, M. & Vanderwert, R. E. Altering facial movements abolishes neural mirroring of facial expressions. Cogn. Affect. Behav. Neurosci. 22, 316–327 (2022).

20. Destrieux, C., Fischl, B., Dale, A. & Halgren, E. Automatic parcellation of human cortical gyri and sulci using standard anatomical nomenclature. NeuroImage 53, 1–15 (2010).

21. Maffei, A. & Sessa, P. Event-related network changes unfold the dynamics of cortical integration during face processing. Psychophysiology 58, e13786 (2021).

22. Maffei, A. & Sessa, P. Time-resolved connectivity reveals the "how" and "when" of brain networks reconfiguration during face processing. Neuroimage Rep. 1, 100022 (2021).

23. Lachaux, J. P., Rodriguez, E., Martinerie, J. & Varela, F. J. Measuring phase synchrony in brain signals. Hum. Brain Mapp. 8, 194–208 (1999).

24. Bruña, R., Maestú, F. & Pereda, E. Phase locking value revisited: teaching new tricks to an old dog. J. Neural Eng. 15, 056011 (2018).

25. Haxby, J. V. & Gobbini, M. I. Distributed neural systems for face perception. In The Oxford Handbook of Face Perception (Oxford Univ. Press, 2011).

26. Quettier, T., Maffei, A., Gambarota, F., Ferrari, P. F. & Sessa, P. Testing EEG functional connectivity between sensorimotor and face processing visual regions in individuals with congenital facial palsy. Front. Syst. Neurosci. 17, 1123221 (2023).

27. Schiano Lomoriello, A. et al. Sensitivity to basic emotional expressions and the emotion perception space in the absence of facial mimicry: the case of individuals with congenital facial palsy. Emotion 24, 602–616 (2024).

28. Wood, A., Lupyan, G., Sherrin, S. & Niedenthal, P. Altering sensorimotor feedback disrupts visual discrimination of facial expressions. Psychon. Bull. Rev. 23, 1150–1156 (2016).

29. Fritz, M. S. & MacKinnon, D. P. Required sample size to detect the mediated effect. Psychol. Sci. 18, 233–239 (2007).
